# Metabolic requirements for cancer cell proliferation

**DOI:** 10.1186/s40170-016-0156-6

**Published:** 2016-08-18

**Authors:** Mark A. Keibler, Thomas M. Wasylenko, Joanne K. Kelleher, Othon Iliopoulos, Matthew G. Vander Heiden, Gregory Stephanopoulos

**Affiliations:** 1 Department of Chemical Engineering, Massachusetts Institute of Technology, Cambridge, MA 02139 USA; 2 Koch Institute for Integrative Cancer Research and Department of Biology, Massachusetts Institute of Technology, Cambridge, MA 02139 USA; 3 Current Address: Late Stage Process Development, Sanofi Genzyme, 31 New York Ave, Framingham, Massachusetts 01701 USA; 4 Center for Cancer Research, Massachusetts General Hospital Cancer Center, Charlestown, MA 02129 USA; 5 Division of Hematology/Oncology, Department of Medicine, Massachusetts General Hospital, Boston, MA 02114 USA

**Keywords:** Cancer metabolism, Proliferation, Biosynthesis, Anabolism, Stoichiometric analysis, Metabolic modeling

## Abstract

**Background:**

The study of cancer metabolism has been largely dedicated to exploring the hypothesis that oncogenic transformation rewires cellular metabolism to sustain elevated rates of growth and division. Intense examination of tumors and cancer cell lines has confirmed that many cancer-associated metabolic phenotypes allow robust growth and survival; however, little attention has been given to explicitly identifying the biochemical requirements for cell proliferation in a rigorous manner in the context of cancer metabolism.

**Results:**

Using a well-studied hybridoma line as a model, we comprehensively and quantitatively enumerate the metabolic requirements for generating new biomass in mammalian cells; this indicated a large biosynthetic requirement for ATP, NADPH, NAD^+^, acetyl-CoA, and amino acids. Extension of this approach to serine/glycine and glutamine metabolic pathways suggested lower limits on serine and glycine catabolism to supply one-carbon unit synthesis and significant availability of glutamine-derived carbon for biosynthesis resulting from nitrogen demands alone, respectively. We integrated our biomass composition results into a flux balance analysis model, placing upper bounds on mitochondrial NADH oxidation to simulate metformin treatment; these simulations reproduced several empirically observed metabolic phenotypes, including increased reductive isocitrate dehydrogenase flux.

**Conclusions:**

Our analysis clarifies the differential needs for central carbon metabolism precursors, glutamine-derived nitrogen, and cofactors such as ATP, NADPH, and NAD^+^, while also providing justification for various extracellular nutrient uptake behaviors observed in tumors. Collectively, these results demonstrate how stoichiometric considerations alone can successfully predict empirically observed phenotypes and provide insight into biochemical dynamics that underlie responses to metabolic perturbations.

**Electronic supplementary material:**

The online version of this article (doi:10.1186/s40170-016-0156-6) contains supplementary material, which is available to authorized users.

## Background

Many parallels exist between the metabolic profiles of cancer cells and normal proliferating cells, including the use of aerobic glycolysis, selective expression of metabolic enzymes with distinct regulatory features, and elevation of amino acid consumption and biosynthesis [1–6]. Growing tumors, like any actively dividing tissue, must continuously generate the precursors for macromolecule synthesis, and if the biomass composition is known, it is possible to determine the minimum rates at which corresponding anabolic substrates must be provided to maintain a specified growth rate. However, unlike many microorganisms, which are capable of synthesizing the entirety of their biomass from a single carbon source and a limited number of salts [7], mammalian cells depend on a complex medium comprised of numerous essential carbon and nitrogen sources [8, 9]. Furthermore, many cancer cells, at least when grown in culture, require a number of nominally nonessential substrates to proliferate (e.g., glutamine and serine), making them conditionally essential for growth [10–13]. Therefore, in culture and also likely in vivo, cancer cells use a variety of nutrients to generate the monomer components of macromolecules, which significantly complicates analysis of their metabolic pathways.

A central aim of the field of cancer metabolism is to identify metabolic pathways selectively activated in tumor cells, which likely include crucial biosynthetic pathways, to reveal therapeutic targets [14, 15]. To accomplish this task, it is necessary to quantify differences in metabolic flux between transformed cells and their differentiated tissues of origin. This can be achieved by direct examination of individual metabolite measurements (e.g., assessing changes in extracellular metabolite concentrations in culture media to calculate consumption and production fluxes; evaluating metabolite pool sizes and enrichments from isotope tracers to indirectly estimate intracellular fluxes [16–18]) or with sophisticated computational approaches in which experimental measurements are incorporated into a data-fitting model to compute a global representation of metabolic behavior (e.g., incorporating extracellular flux and intracellular metabolite isotope labeling data to perform metabolic flux analysis; simulating fluxes in a genome-scale metabolic model constrained by transcriptomic and proteomic data) [19, 20]. However, all of these techniques rely heavily on challenging experimental measurements to infer metabolic trends.

In this investigation, we use fundamental stoichiometric and mass-balance principles to gain insight into the metabolic behavior of cancer cells using only minimal information about their proliferative needs. Previous investigations have similarly used minimal stoichiometric models to explore the effects of using a variety of objective functions on metabolic phenotype, the sensitivity of growth rate and other fluxes to perturbations, and the consistency between these in silico predictions and empirical measurements in a mammalian cell line [21, 22]. Our approach, however, is modeled on more elementary analyses of microbial systems, in which biomass measurements are used to enumerate the corresponding costs in terms of precursors and cofactors [7]. Starting with the well-characterized biochemical composition of hybridoma cells as a model [23], we first give a comprehensive description of all major anabolic requirements for proliferation. Next, using these tabulated requirements as a basis, we perform stoichiometric analyses to identify consequent implications for one-carbon metabolism and glutamine uptake. Finally, we demonstrate how a limited flux balance analysis network can recapitulate observed metabolic behavior with a model for metformin treatment, enabling prediction of cell phenotypes in conditions relevant to cancer through solely stoichiometric principles.

## Methods

### Biomass requirements

#### Weight analysis of biomass composition

**Fig. 1:**
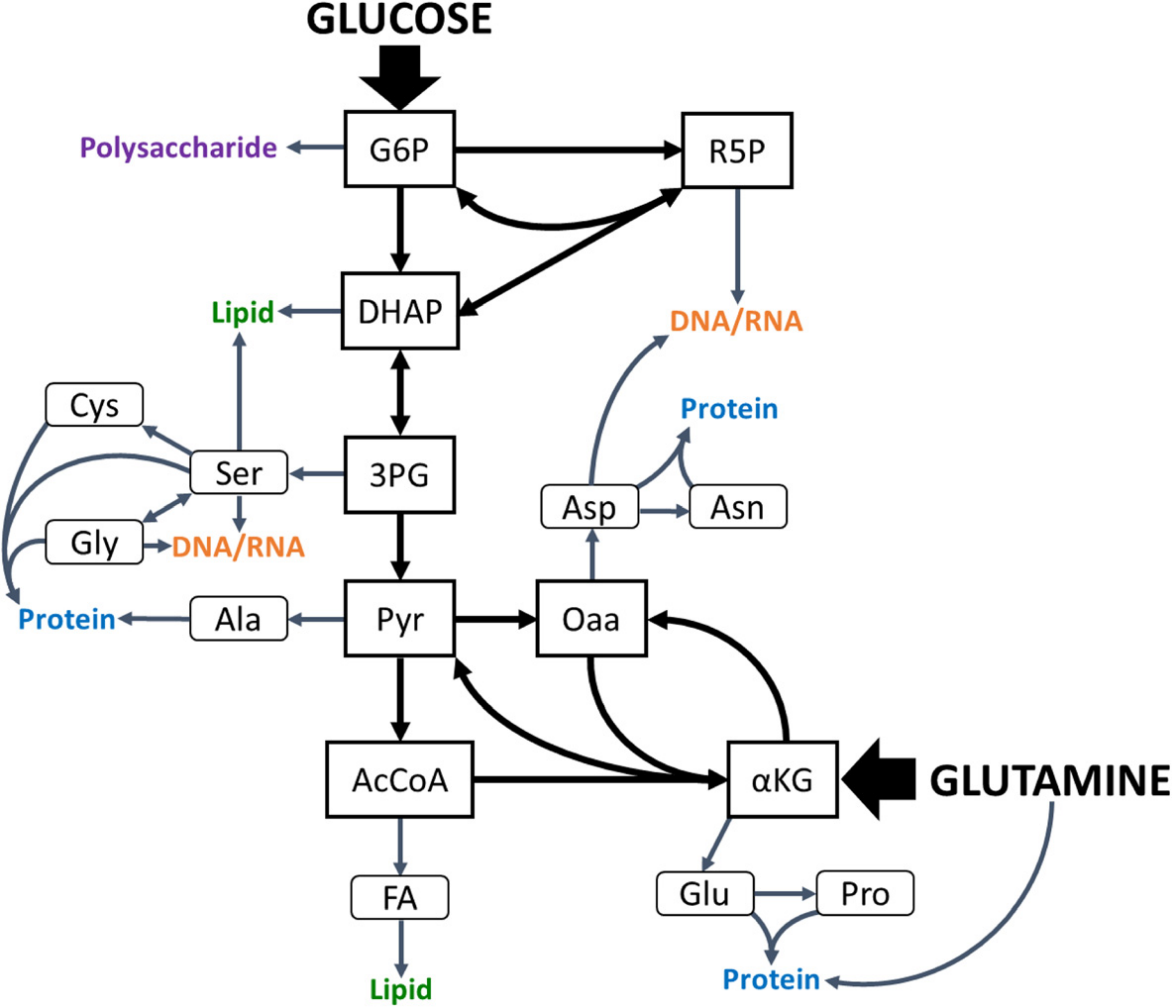
Simplified schematic of central carbon metabolism. *Rectangular boxes* contain branchpoint metabolite intermediates, and *rounded rectangular boxes* contain amino acid and fatty acid products that can be incorporated into biomass macromolecules. *Arrows* indicate carbon flux. Additional metabolic intermediates are not shown; instead, they are implicitly lumped into pools with displayed metabolites (e.g., fructose 6-phosphate with G6P)

Mammalian cell biomass composition was taken from a study that compiled multiple sources of hybridoma biomass composition measurements in the literature [23]. Biomass macromolecules accounted for 962 mg per g dry cell weight (DCW), and this macromolecule fraction was decomposed into its elementary components on a mass basis (Table 1; see Additional file 1: Tables S1–S3 for component masses of each biomass element). “Essential” substrates required for direct extracellular uptake were identified; their weight contributions were consolidated, and their sum was excluded from additional analysis. The remaining “nonessential” components were then decomposed into the anabolic precursors from which they are derived. Eight intermediates in central carbon metabolism where major catabolic pathways diverge into anabolism were designated as carbon sources: glucose 6-phosphate (G6P), ribose 5-phosphate (R5P), dihydroxyacetone phosphate (DHAP), 3-phosphoglycerate (3PG), pyruvate (Pyr), acetyl-coenzyme A (AcCoA), α-ketoglutarate (αKG), and oxaloacetate (Oaa) (Fig. 1) [7, 24]. Nitrogen, phosphorus, and sulfur sources were denoted as NH_3_, PO_4_, and SH, respectively, and the “additional” category represents the contributions of additional inorganic substrates (e.g., water, O_2_, CO_2_) and hydride groups from reduced cofactors. The total mass contribution of each precursor to every macromolecular component was determined (Additional file 1: Tables S1–S3), and these contributions were then scaled by the molar quantities in the given biomass composition to give masses of each per g DCW (Table 1). The “accounted DCW” calculation normalizes DCW percentage values by 962 mg to represent the fractional contribution of each precursor to all components of biomass accounted for by macromolecules. Likewise, the “nonessential component DCW” calculation normalized DCW percentage values by 534 mg, the weight of macromolecular biomass that can be synthesized de novo.

**Table 1:** Composition of 1 g DCW on a precursor mass basis, calculated from hybridoma biomass measurements

Component	Weight per g_DCW_ (mg)	DCW (%)	Accounted DCW (%)	Nonessential component DCW (%)
Essential	428	42.8	44.6	–
G6P	47	4.7	4.9	8.8
R5P	26	2.6	2.7	4.9
DHAP	10	1.0	1.1	1.9
3PG	67	6.7	7.0	12.6
Pyr	33	3.3	3.4	6.1
AcCoA	66	6.6	6.9	12.4
Oaa	64	6.4	6.6	12.0
aKG	99	9.9	10.3	18.6
NH_3_	73	7.3	7.6	13.8
SH	5	0.5	0.5	0.9
PO_3_	28	2.8	2.9	5.3
Additional	14	1.4	1.5	2.6
Total	962	96.2	100	100

Weight fractions are given for total, accounted, and nonessential component DCWs

#### Precursor and cofactor demands

Using the composition of hybridoma cells retrieved from the literature as a basis [23] and scaling by the stoichiometric coefficients in anabolic reactions (Additional file 1: Tables S4–S7 and Supplementary Notes) [8, 25, 26], the molar demands for de novo biomass synthesis were also calculated in units of mmol/g_DCW_ (Table 2). Precursor demands include the eight previously outlined central carbon metabolism intermediates, one-carbon units, and amine groups. Cofactor demands include ATP, NAD^+^, and NADPH. Additionally, molecular oxygen (O_2_) was included. The demands for complete biosynthesis of all nonessential components (i.e., only essential substrates are consumed from the extracellular environment) are listed under the “Synthesis” header. Additionally, these demands were modified to consider the scenario in which nonessential amino acids (NEAAs) and fatty acids (FAs) are consumed from the surrounding medium, with the results listed under the “Uptake” header. (Essential fatty acids such as linoleic and linolenic acids were not explicitly distinguished in the source literature [23] and are therefore not considered separately here.)

**Table 2:** Molar precursor and cofactor demands for producing nonessential biomass components

Precursor/cofactor	Cellular biosynthesis requirements			
	Synthesis of nonessential components		Uptake of nonessential components	
	mmol/g_DCW_	fmol/cell^a^	mmol/g_DCW_	fmol/cell^a^
G6P	0.289	104	0.289	104
R5P	0.233	83.8	0.233	83.8
DHAP	0.119	42.8	0.119	42.8
3PG	1.24	448	0	0
Pyr	0.600	216	0	0
AcCoA	2.46	893	0.324	117
Oaa	0.760	274	0	0
aKG	1.02	368	0	0
1C	0.255	91.8	0.255	91.8
Nitrogen	4.89	1.76 × 10^3^	0.891	321
O_2_	0.387	139	0.198	71.3
NAD^+^	1.19	428	0.0654	23.5
NADPH	5.21	1.88 × 10^3^	0.607	219
ATP	36.0	1.30 × 10^4^	32.1	1.16 × 10^4^

Demands are shown for two separate cases: (1) where all nonessential amino acids and fatty acids are synthesized de novo and (2) where all nonessential amino acids and fatty acids are taken up from the medium. Entries that are identical between the cases indicate that cells cannot substitute nutrient consumption for biosynthesis. Entries that are smaller in “Uptake” than “Synthesis” indicate that cells can substitute nutrient uptake for biosynthesis (by a quantity equal to the difference)

^a^fmol/cell values assume a DCW of 360 pg/cell

We did not incorporate the burden of free ATP when calculating precursor and cofactor demands. Assuming an intracellular concentration of ATP of 4.7 mM [27], a cell volume of 1500 fL [28], and a per-cell dry weight of 360 pg [29] leads to an estimate of 0.0019 mmol free ATP/g_DCW_ that must be synthesized. The corresponding contribution amounts to an addition of roughly 1.5 % to the least abundant quantity (1C) and less than 1 % for other associated components (e.g., R5P, 3PG, nitrogen) in the “Synthesis” regime (Table 2), and we considered these values to be sufficiently small to neglect.

#### Serine, glycine, and one-carbon units

The total serine, glycine, and one-carbon (1C) unit demands per gram DCW were determined by combining the demands for all biomass components for which they serve as substrates (Table 3). 1C units were assumed to be synthesized either from serine catabolism through serine hydroxymethyltransferase (SHMT) or glycine catabolism through the glycine cleavage system (GCS); each pathway was considered separately as the sole source for 1C units, and production through SHMT (GCS) was added to the total demand for serine (glycine). The demands for serine-, glycine-, and 1C-associated biomass (Table 3) were subsequently normalized by *total* (serine/glycine + 1C) serine or glycine demand to give the fractional fate of each amino acid when it serves as the sole source for 1C units (Table 4). Glutathione, which is present at millimolar quantities inside the cell [27], also requires glycine for its synthesis [8]; however, we assumed that, since the original measurements that served as the basis for the tabulated hybridoma composition relied on quantification of total protein levels per cell and protein hydrolysis to give the distribution of amino acids [23, 30], glutathione, as a peptide, has been implicitly considered as proteinogenic glycine.

**Table 3:** Molar requirements of serine, glycine, and one-carbon units for biomass production

Substrate	Fate	mmol/g_DCW_
Serine	Protein	0.43
	Lipid	0.011
	Total	0.441
Glycine	Protein	0.538
	Nucleotide	0.1201
	Total	0.6581
One carbon	Nucleotide	0.255
	Cholesterol Byproduct	−0.018
	Total	0.237

**Table 4:** Fates of serine and glycine

Biomass fate	Biosynthetic pathway	
	SHMT	GCS
Serine	0.65	–
Glycine	–	0.74
One carbon	0.35	0.26

Values represent the fraction of total serine (or glycine) that must be metabolized through SHMT (or the GCS) to meet the demand for one-carbon units

#### Carbon, nitrogen, and glutamine demands

The total cellular carbon and nitrogen molar demands were determined by taking the cumulative sum of all biomass components (mmol/g_DCW_) scaled by their corresponding numbers of carbon and nitrogen atoms, respectively (Table 5 and Additional file 1: Table S8) [23]. Essential demands were determined by taking the cumulative sum of all components that cannot be synthesized de novo (essential amino acids, choline, and ethanolamine). Nonessential carbon and nitrogen demands were determined by subtracting the essential demands from the total demands.

**Table 5:** Glutamine-derived nitrogen and carbon available for biomass contribution

Glutamine uptake profile	Contributed mmol/g_DCW_		Glutamine contribution to total biomass (%)		Glutamine contribution to total nonessential biomass (%)	
	Nitrogen	Carbon	Nitrogen	Carbon	Nitrogen	Carbon
Maximum	4.89	12.2	47.1	29.0	100	57.1
Minimum	1.10	2.75	10.6	6.6	22.5	12.8
Predicted	4.33	10.8	41.6	25.7	88.4	50.5

Biomass contributions are given for maximum, minimum, and predicted glutamine uptake rates. Values are given in mmol/g_DCW_, as well as percentages of total and nonessential biomass

Our analysis assumes that glutamine must be taken up from the media. Glutamine is expected to serve as the primary nitrogen source for macromolecules synthesized de novo [8, 31, 32], and three different regimes of obtaining nitrogenous biomass were considered: (1) a *maximum* uptake profile, where glutamine was assumed to serve as the sole source of nitrogen for synthesizing nonessential molecules; (2) a *minimum* uptake profile, where glutamine was assumed to be consumed only for reactions in which other nitrogen-containing compounds cannot serve as substrates (i.e., proteinogenic glutamine and reactions that consume the amide-amine group); and (3) a *predicted* uptake profile, where glutamine consumption is determined from a simulated stoichiometric network of cells cultured in Dulbecco’s modified Eagle medium (DMEM) (Table 5). For each uptake profile, carbon and nitrogen contributions were calculated by scaling the glutamine consumed by its corresponding number of carbon and nitrogen atoms (five and two, respectively; Additional file 1: Table S8). For each regime, the carbon (nitrogen) contribution from glutamine was divided by the total and nonessential cellular carbon (nitrogen) demands calculated previously to give the fractions of total and nonessential carbon (nitrogen) in biomass predicted to be derived from glutamine (Table 5).

### Flux balance analysis

#### Problem formulation

Flux balance analysis (FBA) [33, 34] was used to generate optimal metabolic flux distributions that maximize growth yield and satisfy stoichiometric constraints on growth. A metabolic model, consisting of 168 metabolites comprising a set ℳ = {1, …, 168} and 152 reactions comprising a set $\mathcal{N}=\left\{1,\dots, 152\right\},$ was used to generate a 168 × 152 stoichiometric matrix. The reactions cover major catabolic and anabolic pathways including glycolysis, the pentose phosphate pathway (PPP), the TCA cycle, the electron transport chain (ETC), one-carbon metabolism, and de novo synthesis of all major macromolecular constituents heretofore considered (Additional file 1: Tables S9, S10). The model is further compartmentalized into mitochondrial and cytosolic pools for metabolites known to engage in distinctly different behavior in each (e.g., cofactors, TCA cycle intermediates), and reactions constituting the malate-aspartate shuttle were included to enable intercompartmental transport of redox species.

This network was incorporated into the following linear programming (LP) problem:

$$ \underset{v}{ \min }Z $$

where

$$ Z={\displaystyle \sum_{n=1}^N}{c}_n{v}_n $$

subject to:

$$ \begin{array}{ll}S\cdot \overset{\rightharpoonup }{v}=\overset{\rightharpoonup }{b}\hfill & \hfill \\ {}{b}_m=0,\hfill & \forall m\in {\mathrm{\mathcal{M}}}_{intr}\hfill \\ {}{b}_m\in \mathrm{\mathbb{R}},\hfill & \forall m\in {\mathrm{\mathcal{M}}}_{extr}\hfill \\ {}{v}_n\ge 0,\hfill & \forall n\in {\mathcal{N}}_{irrev}\hfill \\ {}{v}_n\in \mathrm{\mathbb{R}},\hfill & \forall n\in {\mathcal{N}}_{rev}\hfill \end{array} $$

where *N* = 152 is the total number of reactions, ℳ_intr_ is the set of all intracellular metabolites, ℳ_extr_ is the set of all extracellular metabolites, ${\mathcal{N}}_{\mathrm{irrev}}$ is the set of all irreversible metabolic reactions, and ${\mathcal{N}}_{\mathrm{rev}}$ is the set of all reversible metabolic reactions.

The first constraint is a mass balance; *S* represents the stoichiometric matrix, $\overset{\rightharpoonup }{v}$ represents the flux vector, and $\overset{\rightharpoonup }{b}$ represents the time-derivative vector of metabolite concentrations. The next constraints specify that at steady state, *b*_*m*_ is zero if metabolite *m* is intracellular and *b*_*m*_ is equal to the specific consumption (or production) flux of metabolite *m* if metabolite *m* is extracellular. The final constraints state that irreversible reactions can only take nonnegative values, while reversible reactions can be assigned any real value. Reactions were primarily designated to be irreversible if they were recognized as such by literature and database sources [8, 25, 26]; exceptions were made if a theoretically reversible reaction was known to preferentially operate in a certain direction in cell culture or in vivo (e.g., net secretion of lactate). *Z* is a specified linear combination of metabolic fluxes, which serves as the objective function for the LP problem.

For our metformin treatment simulation (see below), we have chosen to maximize the yield of biomass on carbon. To achieve this, we fixed the specific growth rate as a basis and chose the coefficients *c*_*n*_ such that *Z* is equal to the total net carbon consumption rate:

$$ \mathrm{and}\begin{array}{cc}\hfill \kern1em {c}_n=\frac{\# carbons}{ subs trate},\hfill & \hfill \kern0.75em \forall n\in {\mathcal{N}}_{subs}^{cons}\hfill \\ {}\hfill\ {c}_n=0,\ \hfill & \hfill \kern0.5em \forall n\notin {\mathcal{N}}_{subs}^{cons}\hfill \end{array} $$

where ${\mathcal{N}}_{\mathrm{subs}}^{\mathrm{cons}}$ is the set of all net consumption reactions for glucose, glutamine, all nonessential amino acids, and carbon dioxide. The set of substrates available for uptake was based on the composition of DMEM; therefore, of all nonessential amino acids, only those present in DMEM (cysteine, glycine, and serine) are permitted to have extracellular fluxes in the direction of net consumption.

Biomass composition values were taken from the literature [23] as described above, and specific growth rate was fixed at 0.0289 h^−1^ (i.e., a doubling time of 24 h) [35]. Since growth rate was fixed, substrate consumption rates were left variable and included in the objective functions in the LP problems to find flux distribution solutions that maximized biomass yield on carbon, as described above. For maintenance of ATP cost, we used a literature estimate of 1.55 mmol g_DCW_^−1^ h^−1^ [23]. A DCW of 360 pg/cell was assumed to normalize biomass content on a per-cell basis [29]. Because optimization of growth yield alone cannot capture the Warburg effect [36, 37], a lower limit of lactate production (418 fmol cell^−1^ h^−1^, representative of the highly glycolytic A549 lung carcinoma cell line [16]) was introduced to ensure that the resulting flux distributions reasonably reproduced those empirically observed. (As was the case in previous reports [36, 37], minimization of total carbon alone did not result in lactate production.) To represent the detoxification of reactive oxygen species (ROS), we assumed that 1 % of total oxygen consumed contributes to the formation of ROS, which must be neutralized by one equivalent of mitochondrial NADPH [38].

The minimal carbon uptake rate (i.e., the objective function value Z^*^, which maximizes biomass yields at the specified growth rate) was determined using the “linprog” LP solver function in MATLAB (Version 2009b, Mathworks). However, for a given problem formulation, there are generally multiple flux distributions that have this minimal carbon uptake rate. To avoid the possibility of multiple solutions, we implemented a second optimization program that, in addition to all previously specified constraints, specifies the carbon uptake rate to be equal to the minimal rate Z^*^ (determined from the first problem) and minimizes the two-norm of the flux vector *v*. The complete problem is therefore a bilevel optimization [39] that can be represented by the following formulation:

$$ \underset{v}{ \min}\sqrt{{\displaystyle \sum_{i=1}^N}{v}_i^2} $$

subject to

$$ \underset{v}{ \min }Z $$

subject to:

$$ \begin{array}{ll}S\cdot \overset{\rightharpoonup }{v}=\overset{\rightharpoonup }{b}\hfill & \hfill \\ {}{b}_m=0,\hfill & \forall m\in {\mathrm{\mathcal{M}}}_{intr}\hfill \\ {}{b}_m\in \mathrm{\mathbb{R}},\hfill & \forall m\in {\mathrm{\mathcal{M}}}_{extr}\hfill \\ {}{v}_n\ge 0,\hfill & \forall n\in {\mathcal{N}}_{irrev}\hfill \\ {}{v}_n\in \mathrm{\mathbb{R}},\hfill & \forall n\in {\mathcal{N}}_{rev}\hfill \end{array} $$

#### Metformin treatment simulations

To simulate treatment by metformin, an inhibitor of respiratory complex I, the upper bound of the “ETC_NADH_” reaction, which corresponds to the production of 2.5 equivalents of ATP in exchange for the respiration-linked oxidation of one equivalent of mitochondrial NADH, was successively decreased. Initially, an unconstrained simulation, which used the settings described in the “Problem formulation” section, was performed to give a baseline flux distribution reflecting untreated conditions (0 % nhibition of NADH oxidation by ETC). For all cases that simulated metformin treatment, an upper bound on the ETC_NADH_ flux was introduced. This upper bound was set to 80, 60, 40, 20, and 0 % of the baseline flux value; these conditions were designated 20, 40, 60, 80, and 100 % inhibition of NADH oxidation by ETC, respectively. All output flux distributions are given in Additional file 2: Table S14.

## Results and discussion

### Precursors

**Fig. 2:**
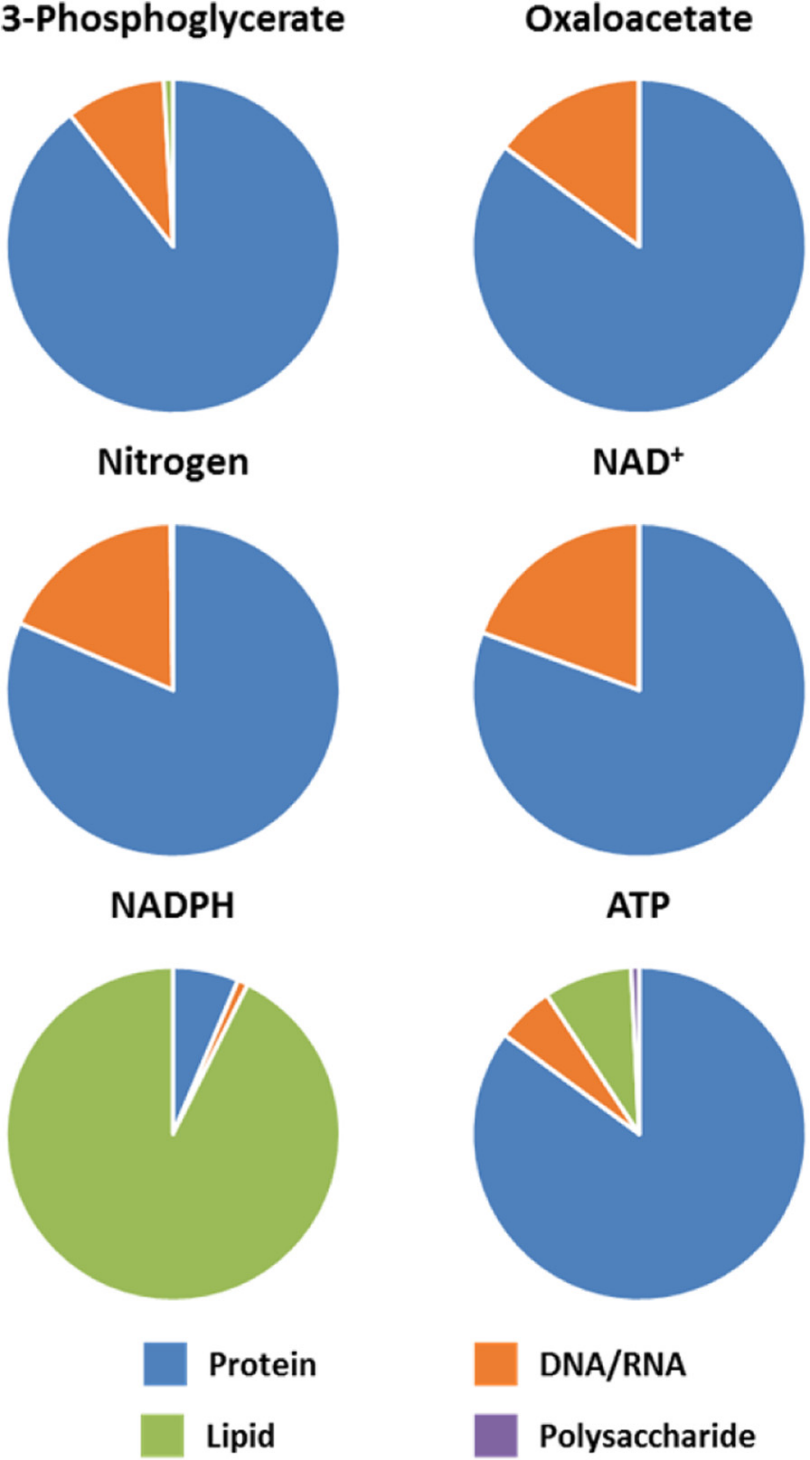
Fates of major biomass precursors and cofactor equivalents consumed in synthesis of macromolecules. Fates of biomass precursors (3-phosphoglycerate and oxaloacetate), nitrogen/amine groups, and cofactors (NAD^+^, NADPH, and ATP) are classified by their requirements for major classes of macromolecules (proteins, nucleotides, lipids, and polysaccharides). Demands for each macromolecule include both costs of polymerization and de novo synthesis of monomers

We obtained a profile of hybridoma composition from Sheikh et al. 2005, which used hydrolyzed biomass data to give an accounting of 96.2 % measured DCW (Table 1). The additional 3.8 %, which presumably consists of small ions, vitamins, and other free metabolites and cofactors, is consistent with other estimates for the DCW fraction comprising free compounds [9, 30, 40]. Of this macromolecular fraction of DCW, essential compounds (i.e., those that must be taken up directly from the surrounding media or serum, such as choline and essential amino acids) constitute close to half—44.6 %. The metabolites in the remaining nonessential fraction can be synthesized de novo from a small set of core central carbon intermediates and other major elemental compounds (i.e., amino, thiol, and phosphate groups), with glucose, glutamine, and other catabolized amino acids as the primary carbon sources. The total demand for each core central carbon metabolite and elemental compound for synthesis of 1 g dry biomass was computed from the hybridoma biomass composition. Not surprisingly, almost all of the largest contributors (i.e., those which contributed at least 10 % of nonessential DCW) were associated with protein (e.g., 3PG, Oaa, αKG, and NH_3_) (Fig. 2), which itself constitutes nearly 75 % of the total DCW. (The exception, AcCoA, is the major precursor of lipids.) No one single precursor contributes more than 10 % of the overall DCW.

In addition to their mass contributions to DCW, we computed the molar requirements of each of the major precursors, 1C units, O_2_, and cofactors such as NAD^+^, NADPH, and ATP, to give the biosynthetic burden of complete de novo genesis of nonessential components (Table 2). The high demand for ATP hydrolysis—at 36.0 mmol/g_DCW_, at least an order of magnitude larger than any other requirement—primarily reflects the significant free energy burden in polymerizing monosaccharides, amino acids, and nucleotides, which alone accounts for an estimated 29.5 mmol/g_DCW_. The values of the next greatest requirements—NADPH and AcCoA and nitrogen, 3PG, and αKG—reflect their substantial involvement in lipid and amino acid synthesis, respectively. (All other components are required at quantities of 1 mmol/g_DCW_ or less, reflecting the relatively low abundance of ribose, polysaccharide, and the glycerol lipid backbone compared to other, mostly protein-associated, components.) It is notable that, in contrast to the use of NADPH for reducing power, NAD^+^ is primarily required for biosynthesis in its oxidized form.

However, this profile assumes complete biosynthesis of all nonessential compounds starting from glucose, whereas in practice, many cells will derive significant contributions to TCA cycle-associated compounds from glutamine and obtain nonessential amino acids and fatty acids directly from surrounding media [32, 41–43]. The effect of such uptake on the biosynthetic burden is shown in Table 2, with considerable reductions in the needs for 3PG, Pyr, Oaa, αKG, and AcCoA, as well as nitrogen (which is largely contributed from glutamine and other nonessential amino acids [32]). Because the majority of ATP equivalents are used for polymerization, which is not influenced by substrate uptake, there is not a substantial reduction in ATP demand. (Although the molar amount of ATP that must be consumed for growth is greater than for all other substrates and cofactors, this quantity is still expected to be less than the ATP maintenance cost for all but the fastest growing cells. Even assuming extracellular uptake of all nonessential biosynthetic substrates, the 1.55 mmol ATP g_DCW_^−1^ h^−1^ expended for cell maintenance estimated for the hybridoma model [23] exceeds growth-associated ATP consumption for doubling times longer than 14.4 h; this doubling time corresponds to faster growth than exhibited by all NCI-60 panel cell lines [35].)

### Serine, glycine, and one-carbon units

A number of recent reports have implicated serine, glycine, and one-carbon metabolism as being important for tumors. The gene for phosphoglycerate dehydrogenase (PHGDH), which encodes the enzyme that catalyzes the first committed step in serine biosynthesis from 3PG, has been found to be amplified in breast cancer and melanoma [11, 12]; glycine consumption and catabolism have been reported to be important for fast proliferation [44, 45]; and oxidation of tetrahydrofolate (THF) compounds has been shown to be used for redox control in cancer cells [18, 46]. In addition, 1C units possess well-established roles in nucleotide synthesis, with methylene-THF required for thymidylate production and its oxidized form, formyl-THF, for purine synthesis.

Nucleotide synthesis is essential for cancer cells, as well as any proliferating cells, to divide. Unlike amino acids (and potentially lipids), which can be derived from serum or culture medium to bypass de novo production [41, 47, 48], nucleotides are not thought to be scavenged from the extracellular environment in sufficient quantities to contribute to growth. Consequently, many classic chemotherapeutic drugs directly inhibit various steps in nucleotide generation, and their administration also induces a range of side effects resulting from impaired proliferation of healthy tissue [14, 49]. Nucleotides are the only major class of macromolecules that require one-carbon THF compounds; accordingly, there is strong motivation to understand the production of one-carbon units in the context of tumor metabolism.

**Fig. 3:**
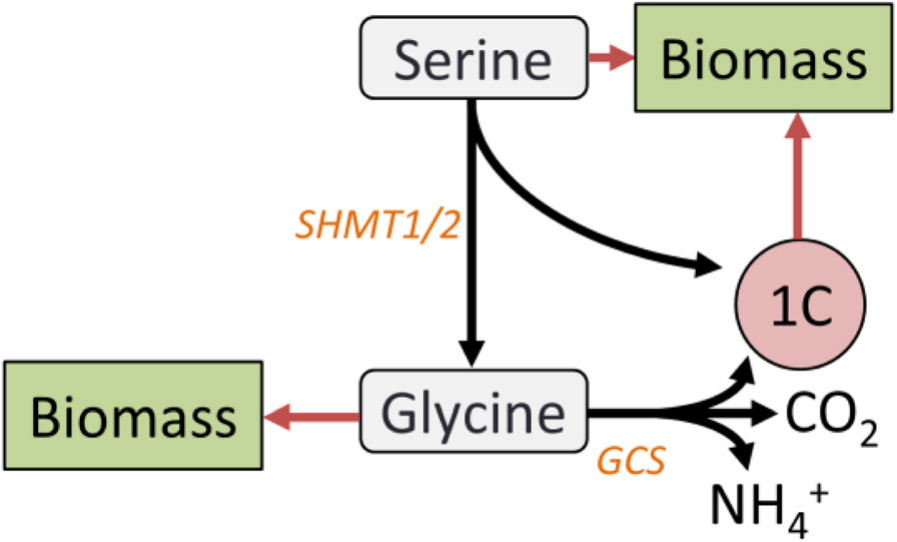
Schematic of the major routes of one-carbon unit production. Serine is catabolized through serine hydroxymethyltransferase (SHMT), and glycine is catabolized through the glycine cleavage system (GCS). Intracellular compartmentalization is not shown

Two major routes exist for methylene-THF generation: SHMT, which couples one-carbon production to serine catabolism to glycine, and the GCS, which oxidizes and deaminates glycine to form methylene-THF, carbon dioxide (CO_2_), and ammonium (NH_4_^+^) (Fig. 3). SHMT has both cytosolic and mitochondrial isoforms—SHMT1 and SHMT2, respectively—while the GCS is exclusively mitochondrial, although intercompartmental transporters exist for serine, glycine, and possibly folates [50, 51].

The demand for 1C units for nucleotide (and potentially NADPH) production places constraints on the fluxes through the serine-glycine pathway. We investigated these constraints by analyzing the biomass requirements of serine, glycine, and tetrahydrofolate compounds. The number of millimoles of each substrate required per gram DCW are given in Table 3; as indicated, the demands for biosynthetic serine and glycine each surpass those of one-carbon units, primarily due to the high requirements for protein synthesis. (Note that each equivalent of cholesterol generated also produces, as a side product, an equivalent of formate which can be directly converted to formyl-THF. The formyl-THF expected to be derived from cholesterol synthesis is subtracted from nucleotide one-carbon requirements.) We considered each extreme case in which 1C substrates are generated exclusively by either serine catabolism via SHMT or glycine catabolism via the GCS, respectively. In the case of SHMT, approximately 1 mol of serine must be metabolized through SHMT to produce 1 mol of 1C for every 2 mol of serine incorporated into biomass (Table 4). In the case of the GCS, it is roughly 1 mol glycine catabolized for every 3 mol glycine incorporated into biomass (Table 4). As noted above, recent studies have suggested that significant 1C production is required for redox control, with SHMT2 being most commonly implicated [18, 45, 46]. If one-carbon units are used for NADPH production for control of oxidative stress in addition to nucleotide synthesis, the 1C demand will increase and the 1:2 and 1:3 ratios of flux through SHMT or GCS to direct biomass incorporation become lower bounds for serine and glycine use; for one-carbon metabolism to contribute significantly to NADPH generation, even larger fractions of the total serine or glycine pool would need to be catabolized through SHMT or the GCS, respectively.

These results give insight into some recent findings about the importance of serine and glycine in the metabolism of cancer cells. Previous results have shown an impairment in proliferation in breast cancer cells with amplified PHGDH copy number when the gene is knocked down, but this knockdown does not result in a change in intracellular serine levels and cannot be rescued by exogenous serine [11]. As in the case where SHMT is used to generate one-carbon units through conversion of serine to glycine, this represents a situation in which *metabolic flux* rather than metabolite levels themselves is important. Although the link between PHGDH and SHMT is less well understood, regulation of biosynthetic pathways at the committed step is a common motif in metabolism, so it is plausible that knockdown of PHGDH may affect the activities of other enzymes in the pathway, including SHMT. Thus, it may be that even when exogenous serine is added to the medium to bypass the PHGDH reaction, the *flux* through the serine-glycine pathway, and therefore production of one-carbon units, is still impaired, this could explain why exogenous serine cannot rescue the PHGDH knockdown.

### Glutamine and nitrogen metabolism

Although glutamine is nominally a nonessential amino acid (it can be synthesized through the ATP-dependent condensation of glutamate and free ammonia), it has been extensively reported that glutamine serves as a major biosynthetic substrate for cancer cells [13, 32, 52]. In effectively all cases that have been examined, cancer cells are not able to proliferate in tissue culture if glutamine is absent, and in particular, expression of the Myc oncogene has been indicated to cause “glutamine addiction,” with glutamine starvation inducing cell death [10, 13, 53, 54]. While glutamine has a unique role in contributing nitrogen to protein and nucleic acid synthesis, its function in maintaining cell viability and division appear to extend beyond this, as its deaminated catabolic product αKG appears at least partially able to rescue survival and/or proliferation under glutamine starvation [55]. Using a stoichiometric analysis, we explored the downstream metabolic consequences of glutamine consumption to satisfy cellular nitrogen demand.

**Fig. 4:**
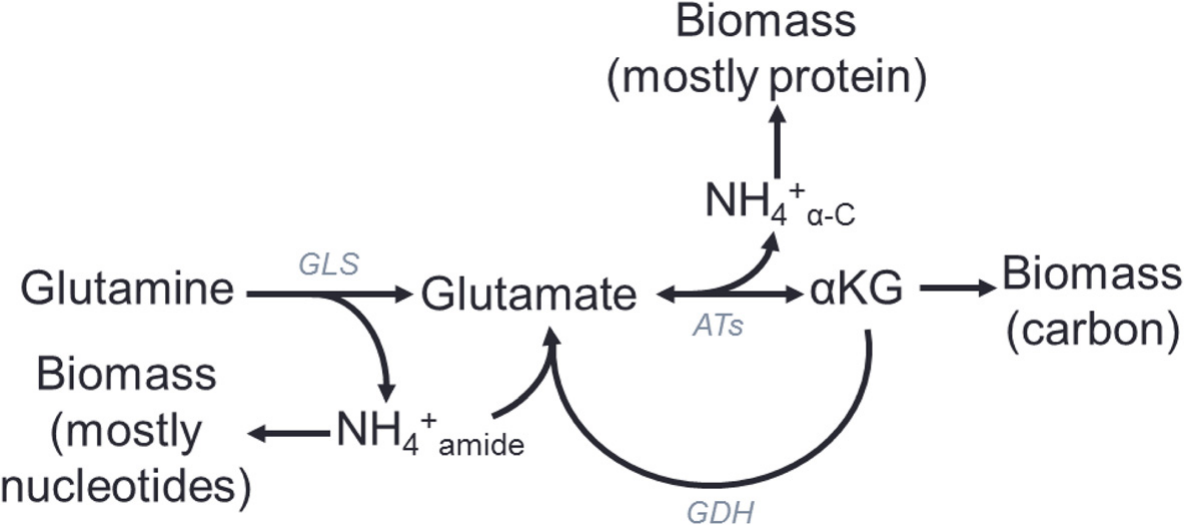
Schematic of the major routes of glutamine contribution to carbon and nitrogen biomass. Deamidation of glutamine to glutamate occurs either via glutaminase (GLS) or various enzymes in nucleotide biosynthesis pathways. Glutamate subsequently can donate its remaining α carbon amine group (NH_4_^+^_α-C_) to α-keto acids via aminotransferases (ATs) to form amino acids, resulting in conversion of the glutamate carbon skeleton to αKG. GLS also produces free ammonium (NH_4_^+^_amide_), which can subsequently be incorporated into αKG to regenerate glutamate by glutamate dehydrogenase (GDH)

Each molecule of glutamine consumed contains two nitrogen atoms that can contribute to biomass generation: an “amide” nitrogen that is lost when glutamine is converted to glutamate and a “transamination” nitrogen that is lost when glutamine-derived glutamate is converted to αKG. Although these two amine groups are used by distinct biosynthesis reactions, the amide nitrogen, for which there is a considerably smaller biomass demand, can be converted to a transamination nitrogen if it is first liberated by glutaminase to become free ammonia and then added to a molecule of αKG by glutamate dehydrogenase to become the amine group in glutamate (Fig. 4). (This model assumes that GDH operates reversibly, which, while thought to be unlikely unless ammonium concentrations are in the range of toxicity, provides a lower bound estimate of potential glutamine contribution to biomass [56].) As a consequence, our analysis does not differentiate between the two amine groups. In addition to its two nitrogen atoms, each glutamine molecule contains five carbon atoms that may be incorporated into biomass precursors, such as AcCoA, αKG, or Oaa. Although assuming that all five carbon atoms may contribute to biomass likely represents an overestimatation due to the presence of several intermediate decarboxylation reactions, which each evolve one carbon as CO_2_, the calculation nonetheless provides a suitable approximation (in addition to the fact that alternate downstream pathways, such as reductive carboxylation of αKG to isocitrate [16, 57, 58], may result in net CO_2_ fixation).

We considered three cases: maximum, minimum, and predicted glutamine contribution to nitrogen supply. The maximum contribution case assumes that glutamine is the only nitrogen source available for incorporation into other nonessential amino acids; the minimum contribution case assumes that glutamine is used only for direct incorporation into protein and donation of its amide group for nucleotide synthesis, with all other nonessential amino acids being taken up directly from the medium; and the predicted contribution case uses the glutamine consumption value obtained from a simulation maximizing biomass yield on total carbon in DMEM nutrient conditions.

The amounts of carbon and nitrogen made available by glutamine uptake under these three scenarios were compared to both the total and nonessential carbon and nitrogen demands for producing new biomass (where “nonessential” designates requirements that can be synthesized de novo) (Additional file 1: Table S8). Consumption of glutamine to meet nitrogen demand can have a profound influence on the supply of biosynthetic carbon: while glutamine contribution under the minimum uptake profile corresponds to a supply of 22.5 % of nonessential nitrogen and 12.8 % of nonessential carbon, its maximum uptake profile corresponds to 100 % of nonessential nitrogen and 57.1 % nonessential carbon (Table 5). The “predicted” uptake profile, in which glutamine supplies all nonessential nitrogen except that needed for cysteine and approximately two thirds of glycine, corresponds very closely to maximal contribution, with 88.4 % of nonessential nitrogen coming from glutamine. In this case, the carbon in the glutamine consumed to meet nitrogen demand is equivalent to roughly half of the total nonessential biosynthetic carbon demand.

These results indicate that proliferating cells may incorporate significant glutamine-derived carbon into the precursors for macromolecular synthesis simply as a consequence of meeting their nitrogen demand. Previous studies indicate that glutamine-consuming cells excrete considerable ammonia and, to a lesser extent, glutamate, which supports the notion of an important role for glutamine beyond nitrogen supply [32, 40, 59, 60]. Whether these findings reflect an involvement in signaling, kinetic/thermodynamic limitations in “efficient” use of nitrogen for anabolism or other metabolic factors remains an open question for the importance of glutamine as a biosynthetic substrate.

### Metformin treatment simulations

Metformin is a safe and widely used biguanide drug that has long been used to treat type II diabetes. Diabetics taking metformin have a reduced incidence of cancer compared to diabetics that control blood sugar by other means, and a surge of investigations has followed to better understand its potential as a cancer therapeutic and its mechanism of action [61–66]. The compound is a direct inhibitor of complex I of the respiratory chain, and although it is believed to trigger numerous downstream phenotypic effects, it also induces substantial short-term, transcription-independent changes in metabolism [67, 68]. These changes reflect the robustness of cell metabolism, and it is important to be able to anticipate these compensatory effects to identify potential routes of adaptation [69, 70]. As with other metabolic inhibitors that may be putatively used as cancer therapeutics, stoichiometric analysis enables the prediction of immediate changes in metabolic fluxes following metformin treatment.

**Fig. 5:**
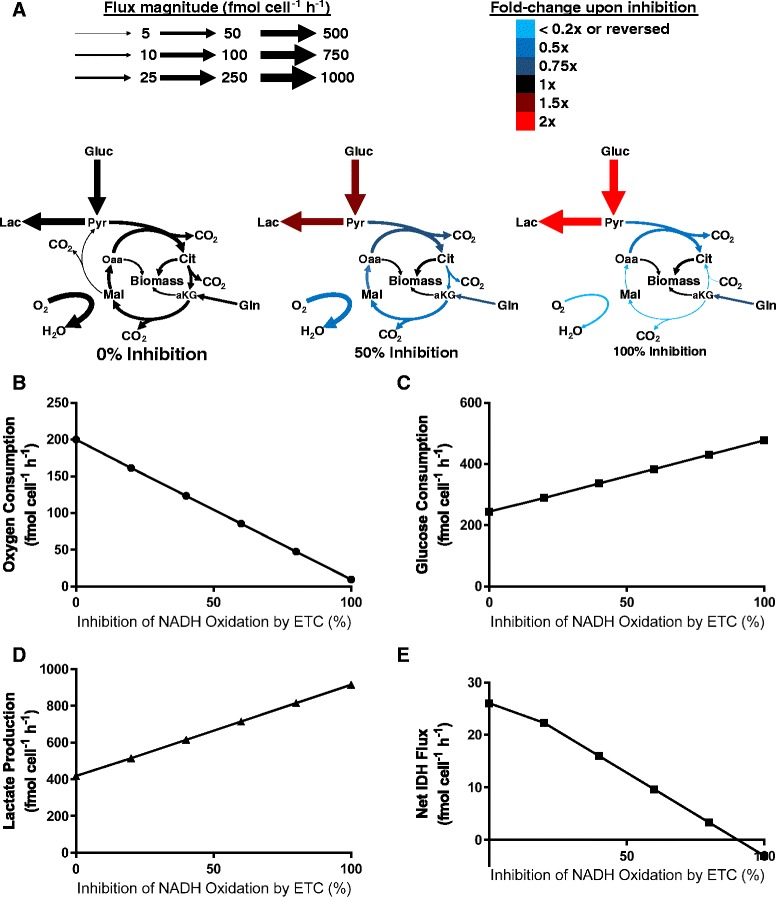
Metabolic flux alterations predicted to occur in response to inhibition of NADH oxidation in the ETC in simulations of metformin treatment. (**a**) Schematic indicating absolute and relative flux values in central carbon metabolism for 0, 50, and 100 % inhibition cases; cellular compartmentalization not shown for simplicity. (**b**) Oxygen consumption, (**c**) glucose consumption, (**d**) lactate production, and (**e**) net IDH fluxes plotted as functions of percent NADH oxidation inhibition

To model the effects of metformin treatment, we applied an FBA approach to a stoichiometric model of central carbon metabolism and major anabolic pathways. After first obtaining a baseline profile of steady-state metabolism of cells, we successively decreased the upper bound on ETC-mediated NADH oxidation to simulate increasing doses of metformin treatment. The flux alterations revealed by these simulations closely mirrored many of the behavioral trends observed experimentally in cancer cells treated with metformin (Fig. 5a; Additional file 1: Tables S11, Additional file 2: Table S14). As would be expected for a respiratory inhibitor, decreasing the upper bound for ETC NADH oxidation reduces the oxygen consumption rate (Fig. 5b); in parallel, glucose consumption and lactate production increase (Fig. 5c, d), presumably to maintain the ATP production rate under effectively anaerobic conditions. Interestingly, increasing levels of inhibition also induce the net direction of the isocitrate dehydrogenase (IDH) reactions to move in the reductive direction, as has been observed in cells treated with metformin and other complex I/III inhibitors (Fig. 5e) [57, 69, 71]. (It should be noted that the net flux considered is the sum of all—mitochondrial and cytosolic—IDH isoform reactions and that net reductive flux is only predicted in the extreme, complete-inhibition case.) While this does not contradict previous findings that reductive IDH flux correlates with a decrease in the citrate-to-αKG ratio [54, 71], it is encouraging that this behavior can be predicted as well in a purely stoichiometric model, which lacks the kinetic and thermodynamic driving forces associated with metabolite concentration changes. To our knowledge, this represents the first instance in which an in silico model has predicted reductive IDH flux following inhibition of mitochondrial NADH oxidation, and it suggests that the phenomenon can be justified in a purely stoichiometric manner.

As previously suggested [69, 71–73], a relative decrease in the mitochondrial NAD^+^ regeneration rate appears to be a factor in the decrease in oxidative TCA cycle flux. ETC NADH oxidation is overwhelmingly the major NAD^+^-producing step in the mitochondria, and constraining this flux correlates closely with decreases in the fluxes through pyruvate dehydrogenase (PDH), oxoglutarate dehydrogenase (OGDH), and mitochondrial malate dehydrogenase (MDH_m_), all of which catalyze NAD^+^-consuming mitochondrial reactions (Additional file 1: Table S12, Additional file 3: Figure S2B–D). While the inhibition of respiration-linked NADH oxidation results in a roughly threefold decrease in total mitochondrial redox activity (i.e., NAD^+^ consumption and production), this is compensated by an approximately twofold increase in total cytosolic redox activity (Additional file 1: Table S13). Primarily, this elevation is accomplished by roughly proportional increases in the major NAD^+^-consuming and NAD^+^-producing reactions in the cytosol, glyceraldehyde 3-phosphate dehydrogenase (GAPDH), and lactate dehydrogenase (LDH), respectively, resulting in an increased fraction of glucose being converted to lactate. In addition, as indicated by lower cytosolic malate dehydrogenase activity upon simulated metformin treatment, malate-aspartate shuttle activity decreased in coordination with lower oxidative TCA cycle flux; this is because NADH generated in glycolysis is oxidized in the cytosol rather than being shuttled into the mitochondria for OXPHOS. In total, these results reflect the tight regulation between redox-associated steps across major metabolic pathways—complex I activity within the ETC, NADH oxidation in the TCA cycle, and GAPDH during and LDH following glycolysis—which, solely by satisfying stoichiometric mass-balance constraints on redox cofactors, enables robust maintenance of cellular growth and homeostasis.

We further explored the issue of NAD^+^ regeneration by assessing the sensitivity of NAD^+^-consuming mitochondrial reactions (PDH, ODGH, and MDH_m_) to the presence of the two non-ETC reactions predicted to oxidize NADH in the mitochondria, nicotinamide nucleotide transhydrogenase (NNT), and NAD^+^-dependent glutamate dehydrogenase (GDH_NAD_). Previous reports have suggested that NNT can promote reductive IDH flux by converting mitochondrial NADH to NADPH [57, 74], and our model predicted NNT to serve as the largest mitochondrial NADH sink under high levels of inhibition and the second-largest mitochondrial NADH sink under conditions of low (or zero) inhibition (Additional file 1: Table S12, Additional file 3: Figure S1A). Removal of NNT from the network produced effectively no changes in any reactions that consumed NAD^+^, but GDH_NAD_, operating in the reverse direction, became the sole reaction aside from ETC NADH oxidation that regenerated mitochondrial NAD^+^, fully compensating for the loss of NNT (Additional file 3: Figure S1B–F, Additional file 2: Table S14). As previously mentioned, GDH activity is not thought to be reversible under baseline conditions of low ammonium concentrations [56], so we repeated our simulations (with and without NNT) after constraining all GDH enzymes to operate irreversibly. The flux distributions that resulted from using the NNT-absent, GDH-irreversible model changed substantially from previous results, with PDH, OGDH, and MDH_m_ fluxes decreasing considerably (Additional file 3: Figure S1C–E, Additional file 2: Table S14). These changes became more dramatic as ETC inhibition increased, with MDH_m_ operating in the negative direction upon high levels of inhibition. Under all conditions, total NADH oxidation was lower than in the original model (Additional file 3: Figure S1F). Inclusion of the NNT reaction in the GDH-irreversible network gave a substantial “rescue” of the metabolic phenotype; NAD^+^-consuming fluxes and total NADH oxidation increased to values much closer to those in the original network (Additional file 3: Figure S1A,C–F, Additional file 2: Table S14). These results support the notion that NNT conversion of NADH to NADPH can substantially contribute to the metabolic phenotype, particularly under conditions of inhibited NADH oxidation by the ETC.

As a final step in the analysis of our metformin simulations, we considered how glutamine usage changed with increasing inhibition of mitochondrial NADH oxidation. Despite dramatic alterations in downstream TCA cycle metabolism [57, 69], glutamine consumption has been reported to either increase slightly or remain unchanged following biguanide treatment [75]. Our simulations provided similar results, predicting a small initial decrease upon constraining ETC NADH oxidation 20 % but no change upon further inhibition (Additional file 3: Figure S2A, Additional file 2: Table S14). However, as previously mentioned, oxidative TCA cycle reactions responsible for catabolizing glutamine-derived α-ketoglutarate decrease (Additional file 3: Figure S2B–D), which is also consistent with previously reported experimental results [57, 71]). These decreases in flux are compensated by increases in reductive carboxylation flux, as noted (Fig. 5e).

To assess the general importance of oxidative glutamine metabolism, we modified our objective function so that glutamine uptake, rather than total carbon uptake, was minimized. As a consequence, glutamine uptake dropped to 11.5 fmol cell^−1^ h^−1^ (reflecting only direct incorporation into protein and contribution of its amide group to asparagine and nucleotide synthesis), and glutaminase flux became zero for all cases (Additional file 2: Table S14), suggesting that oxidative glutaminolysis is not necessary for proliferation. The 7.5 fmol cell^−1^ h^−1^ decrease in glutamine consumption experienced by ETC-inhibited simulations was almost completely offset by a 7.1 fmol cell^−1^ h^−1^ increase in pyruvate carboxylase flux, consistent with reports suggesting that pyruvate carboxylase is required for growth in cells when glutaminase activity is insufficient for anaplerosis [76–78].

Many caveats remain in using this relatively simple FBA model to understand the behavior of cells treated by metformin or, through limiting other fluxes, other metabolic inhibitors. This model assumes that, in response to a particular perturbation, cells are freely able to adjust their metabolic fluxes as necessary to maintain a previously specified growth rate. Cells, of course, are limited in their short-term response to stress by the expression of appropriate enzymes, which involves transcription, translation, and post-translational modifications, with each process responsive on its own distinct time scale. The total cell volume and expression of essential “housekeeping” proteins bound the profile of metabolic enzymes and, hence, fluxes [79]. (Constraining enzyme expression by the total solvent capacity or proteome size is, unlike our approach, able to predict aerobic glycolysis without setting a lower bound on lactate production [36, 37].) Further, signaling cascades (such as the AMPK pathway, which is activated in cells following metformin treatment unless it has been lost [80–82]) are typically triggered by such stresses, and the profile of available fluxes changes following expression of their downstream products. Finally, many of these fluxes, putatively allowed even on the basis of enzyme expression, may be infeasible due to kinetic and thermodynamic constraints, which, aside from the simplified categorization of reactions on the basis of their reversibility, are not captured in this approach. Including additional constraints on fluxes derived from transcriptomic, proteomic, or physicochemical information can overcome some of these limitations and provide more powerful predictive capabilities, but requires a larger, more sophisticated model where such data have been incorporated [19, 36, 37]. While we recognize that it may not be sufficient for some contexts, our approach demonstrates that a relatively small (roughly 150 reaction) stoichiometric network consisting of little more than mass balances, optimized biomass yield, and a lower bound on lactate production is nonetheless capable of predicting metabolic phenotypes in contexts relevant to cancer cells. A comparably simple approach similar to what we pursued here may be more accessible for biologists who do not normally perform computational modeling but are nonetheless interested in simulating metabolic networks to explore their questions.

### Additional discussion

Several additional insights emerge from this analysis. First, on a carbon-molar basis, the fluxes associated with biomass production are small compared to the elevated rates of glucose consumption typical of cancer cells. Considering only the serine biosynthesis pathway and assuming complete de novo synthesis of serine, glycine, and cysteine, cells doubling once per day would require only 41.4 fmol carbon cell^−1^ h^−1^ (Table 2), which would constitute less than 3 % of carbon flux associated with glucose consumption (Additional file 1: Table S11). In fact, at such a growth rate, the carbon required to synthesize the entire nonessential fraction of biomass is equivalent to only roughly 15 % of the carbon intake associated with a typical glucose consumption rate (Additional file 1: Table S8). These numbers suggest that, from a purely stoichiometric or mass-action standpoint, it is very unlikely that the supply of anabolic needs substantially contributes to the largest fluxes in central carbon metabolism in cancer cells (i.e., glycolysis). As would be anticipated given the higher biosynthetic demand for ATP than any biomass building blocks (Table 2), most of the carbon flux is dedicated to producing energy (Additional file 1: Table S11). Indeed, this is consistent with the notion that the distribution of major metabolic fluxes in cancer cells should be insensitive to their specific biomass composition, as has been reported in other studies involving FBA simulations [19]. However, the relatively small magnitudes of these anabolic fluxes do not mean that they are “insignificant” or that they may not serve as promising therapeutic targets for inhibition. Rather, they suggest that additional factors beyond stoichiometry strongly influence the cancer cell metabolic phenotype. Allosteric activation, inhibition, and post-translational modification of enzymes by metabolites or metabolism-mediated epigenetic changes all contribute strongly to metabolic regulation in a complex, bidirectional manner that is difficult to capture using current models [83–86].

Second, the diverse pathways available to cells for cofactor production impart them with enhanced adaptability and robustness in their ability to survive inhospitable microenvironments and potential chemical inhibitors. For the case of ATP, the baseline, uninhibited FBA predicts a roughly 50 % contribution by glycolysis, which is near the upper limit of glycolytic contribution to energy production observed in cancer cells [87] (and largely a result of the assigned lower bound for lactate production). However, the simulated metformin treatment demonstrates how, even approaching complete inhibition of oxidative metabolism, only a roughly twofold increase in fermentation can maintain ATP production without any change in growth. Additionally, numerous pathways exist to allow considerable flexibility for cells to satisfy their demands for NADPH. The FBA model predicts most NADPH to be generated from malic enzyme, glutamate dehydrogenase, nicotinamide nucleotide transhydrogenase, and methylenetetrahydrofolate dehydrogenase, and presumably minimizes flux through the oxidative PPP as a consequence of the objective function (maximized biomass yield on carbon) and lower bound on lactate production. However, given that the oxidative PPP has been demonstrated to possess considerable activity, particularly in response to increased ROS, it too appears to serve a prominent role in generating reducing equivalents in tumor cells [18, 51, 88]. This notion of metabolic flexibility underscores a challenge in targeting cancer cell metabolism, which suggests that combination treatments that minimize the likelihood of adaptation to selective pressure may be promising strategies.

Finally, the high rate of glucose consumption, while enabling the generation of biomass precursors and cofactors beyond necessary requirements, comes at no apparent cost to the cell under these conditions (i.e., the specified growth rate and lactate production rate, each based on tissue culture measurements). Under most in vitro cell culture conditions, glucose concentrations are highly relative to physiological levels (e.g., 25 mM vs. 5–10 mM), and when tumor cells are sufficiently close to blood vessels, they are afforded access to roughly constant glucose levels. In effect, glucose is “free” for these cells, and there is no cost to consuming it when it is available. Although ATP and amino acids are required to generate the enzymes and transporters needed for high rates of fermentation, this burden is presumably considered in the “Protein” biosynthesis demands (Additional file 1: Table S7), and these do not appear prohibitive to growth. Additionally, despite the low yield of ATP per mole glucose consumed, previous analyses using proteomic data have suggested that the overflow metabolism that characterizes the Warburg effect is indeed maximally efficient when considering additional constraints on enzyme levels, such as cellular volume or total protein cost [37, 89, 90]. Furthermore, continuous supply of high glycolytic flux may provide a buffering system such that, in responses to various stresses that may be experienced in the tumor microenvironment (e.g., oxidative stress, energy depletion, drug treatment), flux can be easily shunted to produce substrates (e.g., NADPH, glutathione, ATP) necessary to maintain growth and evade apoptosis.

## Conclusions

In this investigation, we used a series of stoichiometric analyses to elucidate the metabolic requirements of mammalian cell proliferation. First, using a hybridoma line as a model for cancer cell composition, we generated comprehensive profiles of the major precursor and cofactor requirements for biomass synthesis on both mass and stoichiometric bases. These assessments revealed the importance of meeting ATP, NADPH, NAD^+^, and precursor demands in synthesizing new biomass and how these burdens could be selectively reduced by increasing fatty acid and amino acid uptake. Next, we applied the generated profiles to explore the limits of metabolic behavior in two case studies relevant to cancer cells—the production of 1C units from serine and glycine catabolism, and the contribution of glutamine to total cellular nitrogen and carbon—which demonstrate how quantifying biomass demands can yield insight even in the contexts of metabolic branchpoints. These queries demonstrated that flux through serine and glycine biosynthesis pathways is required for sustaining 1C production for nucleotide synthesis and that glutamine may contribute substantially to biomass carbon as a consequence of its natural role as the predominant nitrogen source. Finally, we incorporated these biomass requirements into a constraint-based FBA simulation that modeled the metabolic effects of metformin, a widely used antidiabetic medication currently under consideration as a potential cancer therapeutic. The resulting flux distributions successfully recapitulated the major metabolic changes observed in cells following metformin treatment and also enabled greater understanding of the interactions within the reaction network that contributed to these changes.

## Abbreviations

1C, one-carbon unit; 3PG, 3-phosphoglycerate; αKG, α-ketoglutarate; AcCoA, acetyl-coenzyme A; CS, citrate synthase; DCW, dry cell weight; DHAP, dihydroxyacetone phosphate; DMEM, Dulbecco’s modified Eagle medium; ETC, electron transport chain; FA, fatty acid; FBA, flux balance analysis; FH, fumarate hydratase; G6P, glucose 6-phosphate; GAPDH, glyceraldehyde 3-phosphate dehydrogenase; GCS, glycine cleavage system; GDH, glutamate dehydrogenase; IDH, isocitrate dehydrogenase; LDH, lactate dehydrogenase; LP, linear programming; MDH, malate dehydrogenase; NEAA, nonessential amino acid; NNT, nicotinamide nucleotide transhydrogenase; Oaa, oxaloacetate; OGDH, oxoglutarate dehydrogenase; PHGDH, phosphoglycerate dehydrogenase; PDH, pyruvate dehydrogenase; PPP, pentose phosphate pathway; R5P, ribose 5-phosphate; ROS, reactive oxygen species; SDH, succinate dehydrogenase; SHMT, serine hydroxymethyltransferase; SUCL, succinate-CoA ligase; THF, tetrahydrofolate

## Additional files

Additional file 1: Table S1. Composition of protein biomass components in terms of precursors by weight. **Table S2.** Composition of nucleotide biomass components in terms of precursors by weight. **Table S3.** Composition of polysaccharide and lipid biomass components in terms of precursors by weight. **Table S4.** Stoichiometric precursor and cofactor requirements for nonessential amino acid biosynthesis. **Table S5.** Stoichiometric precursor and cofactor requirements for nucleotide biosynthesis. **Table S6.** Stoichiometric precursor and cofactor requirements for polysaccharide and lipid biosynthesis. **Table S7.** Cumulative stoichiometric precursor and cofactor requirements for major macromolecule groups. **Table S8.** Component breakdown of carbon and nitrogen distribution in biomass. **Table S9.** Metabolites included in the stoichiometric matrix. **Table S10.** Reactions included in the stoichiometric matrix. **Table S11.** Complete flux distributions from metformin treatment simulations. **Table S12.** Mitochondrial NAD^+^-consuming and NAD^+^-producing fluxes computed in metformin treatment simulations. **Table S13.** Cytosolic NAD^+^-consuming and NAD^+^-producing fluxes computed in metformin treatment simulations. (DOCX 121 kb) Additional file 2: Table S14. Flux distributions generated from metformin treatment simulations. Baseline (total carbon uptake minimized, NNT present, GDH reversible); NNT absent, GDH reversible; NNT absent, GDH irreversible; NNT present, GDH irreversible; and total glutamine uptake minimized cases all provided. (XLSX 50 kb) Additional file 3: Figure S1. Mitochondrial NADH consumption and production flux predictions as functions of ETC inhibition for different nicotinamide nucleotide transhydrogenase (NNT) and glutamate dehydrogenase (GDH) conditions. **Figure S2.** Additional metabolic flux alterations predicted to occur in response to ETC inhibition in simulations of metformin treatment. (PDF 194 kb)
