# Supplementary material for: Metabolic requirements for cancer cell proliferation
Source: Cancer Metab. 2016 Aug 18;4:16. doi: 10.1186/s40170-016-0156-6 (PMC4989334; doi:10.1186/s40170-016-0156-6)
Supplement: Additional file 1: Table S1. — Composition of protein biomass components in terms of precursors by weight. Table S2. Composition of nucleotide biomass components in terms of precursors by weight. Table S3. Composition of polysaccharide and lipid biomass components in terms of precursors by weight. Table S4. Stoichiometric precursor and cofactor requirements for nonessential amino acid biosynthesis. Table S5. Stoichiometric precursor and cofactor requirements for nucleotide biosynthesis. Table S6. Stoichiometric precursor and cofactor requirements for polysaccharide and lipid biosynthesis. Table S7. Cumulative stoichiometric precursor and cofactor requirements for major macromolecule groups. Table S8. Component breakdown of carbon and nitrogen distribution in biomass. Table S9. Metabolites included in the stoichiometric matrix. Table S10. Reactions included in the stoichiometric matrix. Table S11. Complete flux distributions from metformin treatment simulations. Table S12. Mitochondrial NAD+-consuming and NAD+-producing fluxes computed in metformin treatment simulations. Table S13. Cytosolic NAD+-consuming and NAD+-producing fluxes computed in metformin treatment simulations. (DOCX 121 kb) [file 40170_2016_156_MOESM1_ESM.docx]

**Supplementary Tables**

**Table S1. Composition of protein biomass components in terms of precursors by weight.**

| **Component** | **Essential** | **3PG** | **Pyr** | **Oaa** | **αKG** | **NH_3_** | **SH** | **Additional** | **Total** |
| --- | --- | --- | --- | --- | --- | --- | --- | --- | --- |
| Ala | - | - | 55.06 | - | - | 15.02 | - | 1.01 | 71.08 |
| Arg | 156.19 | - | - | - | - | - | - | - | 156.19 |
| Asp | - | - | - | 98.06 | - | 15.02 | - | 1.01 | 114.08 |
| Asn | - | - | - | 82.06 | - | 31.04 | - | 1.01 | 114.11 |
| Cys | - | 55.06 | - | - | - | 15.02 | 33.07 | - | 103.14 |
| Gln | - | - | - | - | 96.09 | 31.04 | - | 1.01 | 128.14 |
| Glu | - | - | - | - | 112.08 | 15.02 | - | 1.01 | 128.11 |
| Gly | - | 41.03 | - | - | - | 15.02 | - | 1.01 | 57.05 |
| His | 137.14 | - | - | - | - | - | - | - | 137.14 |
| Ile | 113.16 | - | - | - | - | - | - | - | 113.16 |
| Leu | 113.16 | - | - | - | - | - | - | - | 113.16 |
| Lys | 128.18 | - | - | - | - | - | - | - | 128.18 |
| Met | 131.2 | - | - | - | - | - | - | - | 131.20 |
| Phe | 147.18 | - | - | - | - | - | - | - | 147.18 |
| Pro | - | - | - | - | 80.09 | 14.01 | - | 3.02 | 97.12 |
| Ser | - | 71.055 | - | - | - | 15.02 | - | 1.01 | 87.08 |
| Thr | 101.11 | - | - | - | - | - | - | - | 101.11 |
| Trp | 186.22 | - | - | - | - | - | - | - | 186.22 |
| Tyr | 163.18 | - | - | - | - | - | - | - | 163.18 |
| Val | 99.13 | - | - | - | - | - | - | - | 99.13 |

Values are estimated using hybridoma composition in Sheikh, *et al*., 2005. All units given in mg/g_DCW_.

**Table S2.** Composition of nucleotide biomass components in terms of precursors by weight

| **Component** | **R5P** | **3PG** | **Oaa** | **NH_3_** | **PO_3_** | **Additional** | **Total** |
| --- | --- | --- | --- | --- | --- | --- | --- |
| dAMP | 98.10 | 50.06 | - | 72.05 | 78.97 | 13.02 | 312.20 |
| dCMP | 98.10 | - | 38.05 | 44.04 | 78.97 | 29.02 | 288.17 |
| dGMP | 98.10 | 49.05 | - | 73.06 | 78.97 | 29.02 | 328.20 |
| dTMP | 98.10 | 14.03 | 53.04 | 29.02 | 78.97 | 30.03 | 303.19 |
| AMP | 115.11 | 50.06 | - | 72.05 | 78.97 | 12.01 | 328.20 |
| CMP | 115.11 | - | 38.05 | 44.04 | 78.97 | 28.01 | 304.18 |
| GMP | 115.11 | 49.05 | - | 73.06 | 78.97 | 28.01 | 344.20 |
| UMP | 115.11 | - | 54.05 | 29.02 | 78.97 | 28.01 | 305.16 |

Values are estimated using hybridoma composition in Sheikh, *et al*., 2005. All units given in mg/g_DCW_.

**Table S3.** Composition of polysaccharide and lipid biomass components in terms of precursors by weight.

| **Component** | **Essential** | **G6P** | **DHAP** | **3PG** | **AcCoA** | **NH_3_** | **PO_3_** | **Additional** | **Total** |
| --- | --- | --- | --- | --- | --- | --- | --- | --- | --- |
| Glycogen | - | 162.14 | - | - | - | - | - | - | 162.14 |
| Cholesterol | - | - | - | - | 350.51 | - | - | 36.16 | 386.65 |
| PC | 87.17 | - | 88.06 | - | 486.32 | - | 78.97 | 30.58 | 771.11 |
| PE | 45.09 | - | 88.06 | - | 486.32 | - | 78.97 | 30.58 | 729.02 |
| PI | - | 163.15 | 88.06 | - | 486.32 | - | 78.97 | 30.58 | 847.09 |
| PS | - | - | 88.06 | 71.06 | 486.32 | 17.031 | 78.97 | 30.58 | 772.03 |
| PG | - | - | 163.15 | - | 486.32 | - | 78.97 | 30.58 | 759.03 |
| CL | - | - | 232.19 | - | 972.65 | - | 157.94 | 63.18 | 1425.96 |
| Sphing | 87.17 | - | - | 43.05 | 467.47 | 15.015 | 78.97 | 29.91 | 721.57 |

Values are estimated using hybridoma composition in Sheikh, *et al*., 2005. All units given in mg/g_DCW_.

**Table S4.** **Stoichiometric precursor and cofactor requirements for nonessential amino acid biosynthesis.**

| **Component** | **3PG** | **Pyr** | **Oaa** | **αKG** | **NH_3_** | **SH** | **ATP** | **NADPH** | **NAD^+^** |
| --- | --- | --- | --- | --- | --- | --- | --- | --- | --- |
| Alanine | - | 1 | - | - | 1 | - | - | - | - |
| Aspartate | - | - | 1 | - | 1 | - | - | - | - |
| Asparagine | - | - | 1 | - | 2 | - | 2 | - | - |
| Cysteine | 1 | - | - | - | 1 | 1 | 3 | - | 1 |
| Glutamine | - | - | - | 1 | 2 | - | 1 | - | - |
| Glutamate | - | - | - | 1 | 1 | - | - | - | - |
| Glycine | 1 | - | - | - | 1 | - | - | - | 1 |
| Proline | - | - | - | 1 | 1 | - | 1 | 2 | - |
| Serine | 1 | - | - | - | 1 | - | - | - | 1 |

**Table S5. Stoichiometric precursor and cofactor requirements for nucleotide biosynthesis.**

| **Component** | **R5P** | **3PG** | **Oaa** | **1C** | **NH_3_** | **ATP** | **NADPH** | **NAD^+^** |
| --- | --- | --- | --- | --- | --- | --- | --- | --- |
| dATP | 1 | 1 | - | 2 | 5 | 9 | 1 | 1 |
| dCTP | 1 | - | 1 | - | 3 | 6.5 | 1 | - |
| dGTP | 1 | 1 | - | 2 | 5 | 10 | 1 | 2 |
| dTTP | 1 | - | 1 | 1 | 2 | 7.5 | 1 | - |
| ATP_RNA_ | 1 | 1 | - | 2 | 5 | 9 | - | 1 |
| CTP | 1 | - | 1 | - | 3 | 6.5 | - | - |
| GTP | 1 | 1 | - | 2 | 5 | 10 | - | 2 |
| UTP | 1 | - | 1 | - | 2 | 5.5 | - | - |

Although nucleotides must be activated to the triphosphate form prior to polymerization, only the monophosphate form is physically incorporated into DNA/RNA macromolecules.

**Table S6.** **Stoichiometric precursor and cofactor requirements for polysaccharide and lipid biosynthesis.**

| **Component** | **G6P** | **DHAP** | **3PG** | **AcCoA** | **NH_3_** | **ATP** | **NADPH** | **NAD^+^** | **O_2_** |
| --- | --- | --- | --- | --- | --- | --- | --- | --- | --- |
| Glycogen (monomer) | 1 | - | - | - | - | 1 | - | - | - |
| Cholesterol | - | - | - | 18 | - | 18 | 31 | - | 11 |
| PC | - | 1 | - | 17.43 | - | 22.43 | 32.38 | -1 | 1.52 |
| PE | - | 1 | - | 17.43 | - | 22.43 | 32.38 | -1 | 1.52 |
| PI | - | 1 | - | 17.43 | - | 21.43 | 32.38 | -1 | 1.52 |
| PS | - | 1 | 1 | 17.43 | 1 | 22.43 | 32.38 | - | 1.52 |
| PG | - | 2 | - | 17.43 | - | 21.43 | 32.38 | -2 | 1.52 |
| CL | - | 3 | - | 34.86 | - | 42.86 | 64.76 | -3 | 3.04 |
| Sphing | - | - | 1 | 16.715 | 1 | 21.715 | 32.19 | - | 1.76 |

**Table S7. Cumulative stoichiometric precursor and cofactor requirements for major macromolecule groups.**

| **Component** | | **Protein** | **RNA** | **DNA** | **Lipid** | **Polysaccharide** | **Total** |
| --- | --- | --- | --- | --- | --- | --- | --- |
| **G6P** | | - | - | - | 0.01 | 0.279 | **0.289** |
| **R5P** | | - | 0.1835 | 0.0494 | - | - | **0.233** |
| **DHAP** | | - | - | - | 0.119 | - | **0.119** |
| **3PG** | | 1.113 | 0.0954 | 0.0247 | 0.011 | - | **1.24** |
| **Pyr** | | 0.6 | - | - | - | - | **0.600** |
| **AcCoA** | | - | - | - | 2.4622 | - | **2.46** |
| **Oaa** | | 0.647 | 0.0881 | 0.0247 | - | - | **0.760** |
| **aKG** | | 1.021 | - | - | - | - | **1.02** |
| **1C** | | - | 0.1908 | 0.0642 | - | - | **0.255** |
| **Nitrogen** | | 3.991 | 0.7083 | 0.1828 | 0.011 | - | **4.89** |
| **O_2_** | | - | - | - | 0.3869 | - | **0.387** |
| **NAD^+^** | | 1.113 | 0.1578 | 0.0346 | -0.116 | - | **1.19** |
| **NADPH** | | 0.626 | - | 0.0494 | 4.5392 | - | **5.21** |
| **ATP** | **Monomers** | 1.646 | 1.4607 | 0.4076 | 3.0612 | - | **6.58** |
|  | **Polymerization** | 29.0397 | 0.0734 | 0.0678 | - | 0.279 | **29.5** |
|  | **Total** | 30.6857 | 1.5341 | 0.4753 | 3.0612 | 0.279 | **36.0** |

Values are estimated using hybridoma composition in Sheikh, *et al*., 2005. All units given in mg/g_DCW_.

**Table S8. Component breakdown of carbon and nitrogen distribution in biomass.**

| **Component** | **mmol/ g_DCW_**  [23] | **Atoms Per Component** | | **mmol/g_DCW_** | | | | | |
| --- | --- | --- | --- | --- | --- | --- | --- | --- | --- |
|  |  |  |  | **Total** | | **Essential** | | **Nonessential Uptake (FBA Model)** | |
|  |  | **N** | **C** | **N** | **C** | **N** | **C** | **N** | **C** |
| Ala | 0.6 | 1 | 3 | 0.6 | 1.8 | - | - | - | - |
| Arg | 0.377 | 4 | 6 | 1.508 | 2.262 | 1.508 | 2.262 | - | - |
| Asp | 0.359 | 1 | 4 | 0.359 | 1.436 | - | - | - | - |
| Asn | 0.288 | 2 | 4 | 0.576 | 1.152 | - | - | - | - |
| Cys | 0.145 | 1 | 3 | 0.145 | 0.435 | - | - | 0.145 | 0.435 |
| Gln | 0.322 | 2 | 5 | 0.644 | 1.61 | - | - | - | - |
| Glu | 0.386 | 1 | 5 | 0.386 | 1.93 | - | - | - | - |
| Gly | 0.538 | 1 | 2 | 0.538 | 1.076 | - | - | 0.3443 | 0.6885 |
| His | 0.143 | 3 | 6 | 0.429 | 0.858 | 0.429 | 0.858 | - | - |
| Ile | 0.324 | 1 | 6 | 0.324 | 1.944 | 0.324 | 1.944 | - | - |
| Leu | 0.564 | 1 | 6 | 0.564 | 3.384 | 0.564 | 3.384 | - | - |
| Lys | 0.57 | 2 | 6 | 1.14 | 3.42 | 1.14 | 3.42 | - | - |
| Met | 0.138 | 1 | 5 | 0.138 | 0.69 | 0.138 | 0.69 | - | - |
| Phe | 0.219 | 1 | 9 | 0.219 | 1.971 | 0.219 | 1.971 | - | - |
| Pro | 0.313 | 1 | 5 | 0.313 | 1.565 | - | - | - | - |
| Ser | 0.43 | 1 | 3 | 0.43 | 1.29 | - | - | - | - |
| Thr | 0.386 | 1 | 4 | 0.386 | 1.544 | - | - | - | - |
| Trp | 0.044 | 2 | 11 | 0.088 | 0.484 | - | - | - | - |
| Tyr | 0.182 | 1 | 9 | 0.182 | 1.638 | - | - | - | - |
| Val | 0.416 | 1 | 5 | 0.416 | 2.08 | - | - | - | - |
| dAMP | 0.279 | - | 6 | - | 1.674 | - | - | 0.0095 | 0.0189 |
| dCMP | 0.0148 | 5 | 10 | 0.074 | 0.148 | - | - | - | - |
| dGMP | 0.0099 | 3 | 9 | 0.0297 | 0.0891 | - | - | 0.0063 | 0.0127 |
| dTMP | 0.0099 | 5 | 10 | 0.0495 | 0.099 | - | - | - | - |
| AMP | 0.0148 | 2 | 10 | 0.0296 | 0.148 | - | - | 0.0211 | 0.0422 |
| CMP | 0.033 | 5 | 10 | 0.165 | 0.33 | - | - | - | - |
| GMP | 0.0551 | 3 | 9 | 0.1653 | 0.4959 | - | - | 0.0399 | 0.0799 |
| UMP | 0.0624 | 5 | 10 | 0.312 | 0.624 | - | - | - | - |
| Glycogen (monomer) | 0.033 | 2 | 9 | 0.066 | 0.297 | - | - | - | - |
| Cholesterol | 0.018 | - | 27 | - | 0.486 | - | - | - | - |
| PC | 0.069 | 1 | 42.86 | 0.069 | 2.95734 | 0.069 | 0.345 | - | - |
| PE | 0.026 | 1 | 39.86 | 0.026 | 1.03636 | 0.026 | 0.052 | - | - |
| PI | 0.01 | - | 43.86 | - | 0.4386 | - | - | - | - |
| PS | 0.003 | 1 | 40.86 | 0.003 | 0.12258 | - | - | - | - |
| PG | 0.001 | - | 40.86 | - | 0.04086 | - | - | - | - |
| CL | 0.003 | - | 72.72 | - | 0.21816 | - | - | - | - |
| Sphing | 0.008 | 2 | 43.43 | 0.016 | 0.34744 | 0.008 | 0.04 | - | - |
| **Total** | - | - | - | **10.3901** | **42.1213** | **5.497** | **20.712** | **0.5661** | **1.2772** |

Using total mmol/g_DCW_ and atoms per component, values are computed for total biomass, essential biomass, and nonessential biomass derived from extracellular uptake (as predicted by an FBA model).

**Table S9. Metabolites included in the stoichiometric matrix.**

**Table S10. Reactions included in the stoichiometric matrix.**

**Table S11.** **Complete flux distributions from metformin treatment simulations.**

See end of manuscript for Tables S9-S11.

**Table S12.** **Mitochondrial NAD^+^-consuming and -producing fluxes computed in metformin treatment simulations.**

|  | **Inhibition of NADH Oxidation by ETC** | | | | | |
| --- | --- | --- | --- | --- | --- | --- |
| **Flux** | **0%** | **20%** | **40%** | **60%** | **80%** | **100%** |
| *Consumption* |  |  |  |  |  |  |
| PDH | 51.65 | 47.90 | 41.56 | 35.23 | 28.90 | 22.56 |
| OGDH | 37.62 | 30.64 | 24.31 | 17.97 | 11.64 | 5.304 |
| MDH_m_ | 99.06 | 81.88 | 69.21 | 56.54 | 43.87 | 31.21 |
| **Total** | **188.3** | **160.4** | **135.1** | **109.7** | **84.40** | **59.08** |
| *Production* |  |  |  |  |  |  |
| ETC_NADH_ | 156.5 | 125.2 | 93.87 | 62.58 | 31.29 | 0 |
| GDH_NAD_ | 14.22 | 17.018 | 16.26 | 18.20 | 20.55 | 22.53 |
| NNT | 17.66 | 18.23 | 24.95 | 28.96 | 32.57 | 36.55 |
| **Total** | **188.3** | **160.4** | **135.1** | **109.7** | **84.40** | **59.08** |

All units in fmol cell^-1^ h^-1^.

**Table S13.** **Cytosolic NAD^+^-consuming and -producing fluxes computed in metformin treatment simulations.**

|  | **Inhibition of NADH Oxidation by ETC** | | | | | |
| --- | --- | --- | --- | --- | --- | --- |
| **Flux** | **0%** | **20%** | **40%** | **60%** | **80%** | **100%** |
| *Consumption* |  |  |  |  |  |  |
| GAPDH | 478.7 | 567.9 | 662.0 | 756.0 | 850. 1 | 944.1 |
| PHGDH | 6.450 | 0 | 0 | 0 | 0 | 0 |
| GTP | 0.6492 | 0.6492 | 0.6492 | 0.6492 | 0.6492 | 0.6492 |
| dGTP | 0.103 | 0.103 | 0.103 | 0.103 | 0.103 | 0.103 |
| **Total** | **485.9** | **568.6** | **662.7** | **756.8** | **850.8** | **944.9** |
| *Production* |  |  |  |  |  |  |
| LDH | 418 | 514.2 | 614.6 | 715.0 | 815.4 | 915.8 |
| MDH_c_ | 63.37 | 49.94 | 43.61 | 37.27 | 30.94 | 24.62 |
| P5CR_NAD_ | 3.257 | 3.257 | 3.257 | 3.257 | 3.257 | 3.246 |
| DAG | 1.03 | 1.03 | 1.03 | 1.03 | 1.03 | 1.03 |
| CDP-DAG | 0.1665 | 0.1665 | 0.1665 | 0.1665 | 0.1665 | 0.1665 |
| PGly | 0.0104 | 0.0104 | 0.0104 | 0.0104 | 0.0104 | 0.0104 |
| CL | 0.0312 | 0.0312 | 0.0312 | 0.0312 | 0.0312 | 0.0312 |
| **Total** | **485.9** | **568.6** | **662.7** | **756.8** | **850.8** | **944.9** |

All units in fmol cell^-1^ h^-1^.

**Supplemental Notes**

To determine the molar precursor and cofactor requirements for *de novo* biomass synthesis (given in Tables S4-S6), stoichiometric relationships were obtained from the literature, and a set of assumptions were made to reduce the associated substrates to a common biochemical currency. These foundational assumptions are given below, followed by a detailed description of how they were used to determine the requirements of each biomass component.

**Assumptions**

1. “ATP” refers primarily to the free energy change associated with the group transfer of the γ-phosphate from ATP onto another metabolite to give ADP and P_i_, i.e.

The reaction

1. ATP 🡪 ADP + P_i_

is designated “ATP” for simplicity (i.e. the products ADP and P_i_ are not included). This reaction corresponds to the group transfer and subsequent displacement of a phosphoryl group; however, in some cases, a pyrophosphoryl, adenylyl, or adenosyl group is transferred and displaced instead:

1. ATP 🡪 AMP + PP_i_
2. ATP 🡪 adenosine + PP_i_ + P_i_

Because these exergonic processes are coupled to endergonic reactions to make them thermodynamically feasible, they are quantified roughly by free energy changes under standard biochemical conditions (ΔG’°). Reaction (i), designated “ATP,” possesses a ΔG’° of -30.5 kJ/mol. Reaction (ii), which is also accompanied by the spontaneous hydrolysis of pyrophosphate (PP_i_ 🡪 2P_i_), possesses a total ΔG’° of -64.8 kJ/mol and is taken as “2ATP.” Reaction (iii), which is also accompanied by pyrophosphate hydrolysis, possesses a total ΔG’° of -79 kJ/mol and is approximated as “3ATP.”

1. This tabulation only represents the *costs* associated with synthesizing major biomass components. It is not intended to consider metabolites produced during metabolism of additional byproducts, which may introduce unspecified degrees of freedom in our present analysis. (For instance, the production of cysteine from methionine and serine is associated with α-ketobutyrate, which can be either secreted or further catabolized to generate succinyl-CoA, which itself can be processed in different ways.) In these cases, byproducts are excluded from further consideration, as indicated by strikethrough items. (These byproducts are included in the full stoichiometric network used to assess the metabolic response to metformin treatment.)
2. Although they are primarily generated through serine and glycine metabolism (and would therefore require the costs associated with serine and glycine synthesis unless serine and glycine are taken up from the culture medium), 1C units, which possess multiple routes of production, are considered a distinct category of precursor requirement. (Their production is considered separately.)
3. Due to their abundance, H_2_O and CO_2_/HCO_3_^-^ are not considered as requirements, and they are removed from reactions where they appear as products or reactants.
4. Notation for NADPH and NAD^+^ reactions is simplified as follows:
   - “NADPH” is considered equivalent to NADPH + H^+^ 🡪 NADP^+^ + 2H^+^ + 2e^-^
   - “NAD^+^” is considered equivalent to NAD^+^ + 2H^+^ + 2e^-^ 🡪 NADH + H^+^
   - “(-1)NAD^+^” is considered equivalent to NADH + H^+^ 🡪 NAD^+^ + 2H^+^ + 2e^-^

**Amino Acids**

**Alanine:**

- Pyr + Glu 🡪 Ala + αKG
  - Glu is considered to be αKG and a NH_4_^+^_α-C_.
- **Pyr + NH_4_^+^_α-C_** 🡪 **Ala**

**Aspartate:**

- Oaa + Glu 🡪 Asp + αKG
  - Glu is considered to be αKG and a NH_4_^+^_α-C_.
- **Oaa + NH_4_^+^_α-C_ 🡪 Asp**

**Asparagine:**

- Asp + Gln + ATP 🡪 Asn + Glu + AMP + PP_i_
  - ATP conversion to ADP and PP_i_, is considered “2ATP.”
  - Gln is considered to be Glu and a NH_4_^+^_amide_.
  - Asp is considered to be Oaa and a NH_4_^+^_α-C_.
- **Oaa + NH_4_^+^_α-C_ + NH_4_^+^_amide_ + 2ATP 🡪 Asn**

**Cysteine:**

- Ser + Met + ATP + R 🡪 Cys + NH_4_^+^ + αKb + ade + PPi + Pi + R-CH3
  - Met contributes a thiol group to cysteine production; the associated methyl group is given to an unspecified acceptor, the amino group becomes free NH_4_^+^, and the remaining carbon skeleton is converted to αKb. These additional byproducts are not considered.
  - ATP conversion to adenosine, PP_i_, and P_i_ is considered “3ATP.”
  - Biosynthesis of Ser requires 3PG, NAD^+^, and a NH_4_^+^_α-C_.
  - Simplify NAD^+^ notation
- Ser + SH + ATP 🡪 Cys + ade + PP_i_ + P_i_
- Ser + SH + 3ATP 🡪 Cys
- **3PG + NAD^+^ + NH_4_^+^_α-C_ + SH 🡪 Cys**

**Glutamine**

- αKG + R-NH_4_^+^_α-C_ + NH_4_^+^ + ATP 🡪 Gln + R + ADP + P_i_
  - R-NH_4_^+^ _α_ is an amino acid and R is a keto acid. Glu is considered to be αKG and a NH_4_^+^_α-C_.
  - Glutamine synthetase catalyzes the ATP-dependent condensation of free NH_4_^+^ and Glu to give Gln. The free NH_4_^+^ constitutes the NH_4_^+^_amide_ of Gln.
- **αKG + NH_4_^+^_α-C_ + NH_4_^+^_amide_ + ATP 🡪 Gln**

**Glutamate**

- αKG + R-NH_4_^+^_α-C_ 🡪 Glu + R
  - R-NH_4_^+^ _α_ is an amino acid and R is a keto acid. Glu is considered to be αKG and a NH_4_^+^_α-C_.
- **αKG + NH_4_^+^_α-C_ 🡪 Glu**

**Glycine**

- Ser + THF 🡪 Gly + CH_2_-THF
  - Biosynthesis of Ser requires 3PG, NAD^+^, and a NH_4_^+^_α-C_.
  - Production of CH_2_-THF, a 1C byproduct, is not considered. THF, a carrier for 1C units, is also not considered.
- 3PG + NAD^+^ + NH_4_^+^_α-C_ + THF 🡪 Gly + CH_2_-THF
- **3PG + NAD^+^ + NH_4_^+^_α-C_** 🡪 **Gly**

**Proline**

- Glu + ATP + 2NAD(P)H 🡪 Pro + ADP + P_i_
  - Assume all reducing power comes from NADPH
  - Simplify ATP notation
- **αKG +** **NH_4_^+^_α-C_ + ATP + 2NADPH** 🡪 **Pro**

**Serine**

- 3PG + NAD^+^ + Glu + 🡪 Ser + αKG
  - Glu is considered to be αKG and a NH_4_^+^_α-C_.
- **3PG + NAD^+^ + NH_4_^+^_α-C_ + 🡪 Ser**

**Nucleotides**

1. PRPP Synthesis

- R5P + ATP 🡪 PRPP + AMP + PP_i_
  - ATP conversion to ADP and PP_i_, is considered “2ATP.”
- R5P + 2ATP 🡪 PRPP

**Purine Synthesis**

1. IMP Synthesis

- PRPP + 2Gln + Gly + 2CHO-THF + Asp + 4ATP + CO_2_ 🡪 IMP + 2Glu + PP_i_ + 2THF + 4(ADP + P_i_) + Fum
  - Simplify 1C and ATP notation; remove CO_2_
  - Biosynthesis of Gly requires 3PG, NAD^+^, and a NH_4_^+^_α-C_.
  - Gln is considered to be Glu and a NH_4_^+^_amide_.
  - Asp is considered to be Fum and a NH_4_^+^ _α-C_.
  - Eliminate PP_i_
  - Substitute for PRPP
- PRPP + 2NH_4_^+^_amide_ + 3PG + NAD^+^ + 2NH_4_^+^_α-C_ + 2(1C) + 4ATP 🡪 IMP
- R5P + 2NH_4_^+^_amide_ + 3PG + NAD^+^ + 2NH_4_^+^_α-C_ + 2(1C) + 6ATP 🡪 IMP

1. ATP_RNA_ Synthesis

- IMP + GTP + Asp + 2ATP 🡪 ATP_RNA­_ + (GDP + P_i_) + Fum + 2(ADP + P_i_)
  - Asp is considered to be Fum and a NH_4_^+^ _α-C_.
  - GTP hydrolysis is considered equivalent to ATP hydrolysis; simplify notation.
  - Substitute for IMP
- IMP + 2NH_4_^+^_α-C_ + 3ATP 🡪 ATP_RNA­_
- **R5P + 3NH_4_^+^_α-C_ + 2NH_4_^+^_amide_ + 3PG + NAD^+^ + 2(1C) + 9ATP 🡪 ATP_RNA­_**

1. GTP Synthesis

- IMP + NAD^+^ + Gln + 3ATP 🡪 GTP + Glu + AMP + PP_i_ + 2(ADP + P_i_)
  - ATP conversion to ADP and PP_i_, is considered “2ATP.”
  - Gln is considered to be Glu and a NH_4_^+^_amide_.
  - Simplify ATP notation
  - Substitute for IMP
- IMP + NAD^+^ + NH_4_^+^_amide_ + 4ATP 🡪 GTP
- **R5P + 2NH_4_^+^_α-C_ + 3NH_4_^+^_amide_ + 3PG + 2NAD^+^ + 2(1C) + 10ATP 🡪 GTP**

**Pyrimidine Synthesis**

1. Dihydroorotate synthesis

- HCO_3_^-^ + 2ATP + Gln + Asp 🡪 dihydroorotate + 2(ADP + P_i_) + Glu
  - Remove HCO_3_^-^
  - Gln is considered to be Glu and a NH_4_^+^_amide_.
  - Asp is considered to be Oaa and a NH_4_^+^_α-C_.
  - Simplify ATP term
- 2ATP + Oaa + NH_4_^+^_α-C_ + NH_4_^+^_amide_ 🡪 dihydroorotate

1. Dihydroorotate to orotate conversion

- dihydroorotate + Q 🡪 orotate + QH_2_
  - Ubiquinol (QH_2_) formation is associated with translocation of 2H^+^ and therefore generation of 0.5ATP via the ETC (4H^+^ translocated = 1 ATP)
  - Substitute for dihydroorotate
- dihydroorotate 🡪 orotate + 0.5ATP
- 1.5ATP + Oaa + NH_4_^+^_α-C_ + NH_4_^+^_amide_ 🡪 orotate

1. UMP Synthesis

- orotate + PRPP 🡪 UMP + PP_i_ + CO_2_
  - Remove PP_i_ and CO_2_
  - Substitute for orotate and PRPP
- R5P + 3.5ATP + Oaa + NH_4_^+^_α-C_ + NH_4_^+^_amide_ 🡪 UMP

1. UTP Synthesis
   - 1. UMP phosphorylation by nucleoside phosphate kinases to UTP
     2. Simplify ATP terms
     3. Substitute for UMP
   1. UMP + 2ATP 🡪 UTP + 2(ADP + P_i_)
   2. **R5P + 5.5ATP + Oaa + NH_4_^+^_α-C_ + NH_4_^+^_amide_ 🡪 UTP**
2. CTP Synthesis

- UTP + Gln + ATP 🡪 CTP + Glu + ADP + P_i_
  - Simplify ATP terms
  - Gln is considered to be Glu and a NH_4_^+^_amide_.
  - Substitute for UTP
- UTP + Gln + ATP 🡪 CTP + Glu + ADP + P_i_
- **R5P + 6.5ATP + Oaa + NH_4_^+^_α-C_ + 2NH_4_^+^_amide_ 🡪 CTP**

**dNTP Synthesis**

1. dNTP synthesis from NTPs (dATP, dCTP, dGTP)

- NTP + NADPH 🡪 dNTP (for N = A, C, G)
  - Substitute for NTPs
- **R5P + 3NH_4_^+^_α-C_ + 2NH_4_^+^_amide_ + 3PG + NAD^+^ + 2(1C) + 9ATP + NADPH 🡪 dATP_­_**
- **R5P + 6.5ATP + Oaa + NH_4_^+^_α-C_ + 2NH_4_^+^_amide_ + NADPH 🡪 dCTP**
- **R5P + 2NH_4_^+^_α-C_ + 3NH_4_^+^_amide_ + 3PG + 2NAD^+^ + 2(1C) + 10ATP + NADPH 🡪 dGTP**

1. UMP conversion to UDP
   1. UMP + ATP 🡪 UDP + ADP
   - Simplify ATP term

- UMP + ATP 🡪 UDP

1. UDP conversion to dUDP
   1. UDP + NADPH + H^+^ 🡪 dUDP + NADP^+^
      1. Simplify NADPH term
      2. Substitute for UDP
   2. UMP + ATP + NADPH 🡪 dUDP
2. dUDP conversion to dUTP
   1. dUDP + ATP 🡪 dUTP + ADP
      1. Simplify ATP term
      2. Substitute for dUDP
   2. UMP + 2ATP + NADPH 🡪 dUTP
3. dUTP conversion to dUMP
   1. dUTP + H_2_O 🡪 dUMP + PP_i_
      1. Remove H_2_O and PP_i_ terms
      2. Substitute for dUTP
   2. UMP + 2ATP + NADPH 🡪 dUMP
4. dUMP conversion to dTMP
   1. dUMP + CH_2_-THF 🡪 dTMP + DHF
      1. Simplify 1C notation
      2. Substitute for dUMP
   2. UMP + 2ATP + NADPH + 1C 🡪 dTMP
5. dTTP Synthesis
   1. dTMP + 2ATP 🡪 dTTP + 2(ADP + P_i_)
      1. Simplify ATP term
      2. Substitute for dTMP and UMP
   2. UMP + 4ATP + NADPH + 1C 🡪 dTTP
   3. **R5P + 7.5ATP + Oaa + NH_4_^+^_α-C_ + NH_4_^+^_amide_ + NADPH + 1C 🡪 dTTP**

**Lipids**

1. Fatty acid synthesis (length of n carbons with u unstaturated bonds)
   1. AcCoA and ATP terms simplified:

- $\left( \frac{n}{2} \right)AcCoA+\left( \frac{n}{2}-1 \right)ATP+\left( n+u-2 \right)NADPH+uO_{2}\to{FA}_{n:u}$

1. Average fatty acid composition: n = 17.43, u = 0.76 (Sheikh *et al.*, 2005)

- 8.715AcCoA + 7.15ATP + 16.19NADPH + 0.76O_2_ 🡪 FA

1. Palmitate: n = 16, u = 0

- 8AcCoA + 7ATP + 14NADPH 🡪 Palm

1. Fatty acid activation to acyl-CoA

- FA_n:u_ + ATP + CoA 🡪 FA_n:u_-CoA + AMP + PP_i_
  - ATP conversion to ADP and PP_i_, is considered “2ATP.”
  - Remove CoA term.

FA_n:u_ + 2ATP 🡪 FA_n:u_-CoA + AMP + PP_i_

1. Acyl-CoA Synthesis (Substitute FA and Palm)
2. Average fatty acyl-CoA

- 8.715AcCoA + 9.715ATP + 16.19NADPH + 0.76O_2_ 🡪 FA-CoA

1. Palmitoyl-CoA

- 8AcCoA + 9ATP + 14NADPH 🡪 Palm-CoA

1. 1,2-diacylglycerol Synthesis

- DHAP + NADH + 2FA-CoA 🡪 1,2-DAG + P_i_ + 2CoA
  - Rearrange NAD^+^ term.
  - Remove P_i_ and CoA terms
  - Substitute for FA-CoA
- DHAP + (-1)NAD^+^ + 2FA-CoA 🡪 1,2-DAG
- DHAP + (-1)NAD^+^ + 17.43AcCoA + 19.43ATP + 32.38NADPH + 1.52O_2_ 🡪 1,2-DAG

1. CDP-DAG Synthesis
   1. DHAP + NADH + 2FA-CoA + CTP 🡪 CDP-DAG + PP_i_ + 2CoA
   2. GTP hydrolysis is considered equivalent to ATP hydrolysis; simplify notation.
      1. CTP 🡪 CDP-R + PP_i_ group transfer is considered equivalent to “2ATP.”
      2. Rearrange NAD^+^ term
      3. Remove CoA term
      4. Reduce terms and replace CTP with ATP terms
   3. DHAP + (-1)NAD^+^ + 2FA-CoA + 2ATP 🡪 CDP-DAG
   4. DHAP + (-1)NAD^+^ + 17.43AcCoA + 21.43ATP + 32.38NADPH + 1.52O_2_ 🡪 CDP-DAG
2. **Phosphotidylcholine Synthesis**

- choline + ATP + CTP + 1,2-DAG 🡪 PC + ADP + CMP + PP_i_
  - CTP 🡪 CMP + PP_i_ hydrolysis is considered equivalent to “2ATP.”
  - Substitute for 1,2-DAG
- choline + 3ATP + 1,2-DAG 🡪 PC
- **DHAP + (-1)NAD^+^ + 17.43AcCoA + 22.43ATP + 32.38NADPH + 1.52O_2_ + choline 🡪 PC**

1. **Phosphotidylethanolamine Synthesis**
   1. EA + ATP + CTP + 1,2-DAG 🡪 PE + ADP + CMP + PP_i_
      1. CTP 🡪 CMP + PP_i_ hydrolysis is considered equivalent to “2ATP.”
      2. Substitute for 1,2-DAG
   2. **DHAP + (-1)NAD^+^ + 17.43AcCoA + 22.43ATP + 32.38NADPH + 1.52O_2_ + EA 🡪 PE**
2. **Phosphotidylglycerol Synthesis**

- DHAP + NADH + CTP + 1,2-DAG 🡪 PG + CMP + PP_i_ + P_i_
  - Rearrange NAD^+^ term.
  - CTP 🡪 CMP + PP_i_ hydrolysis is considered equivalent to “2ATP.”
  - Remove P_i_ product.
  - Substitute 1,2-DAG term.
- DHAP + (-1)NAD^+^ + 2ATP + 1,2-DAG 🡪 PG
- **2DHAP + (-2)NAD^+^ + 17.43AcCoA + 21.43ATP + 32.38NADPH + 1.52O_2_ 🡪 PG**

1. **Phosphatidylserine Synthesis**

Two synthetic routes are available:

1. PC + Ser 🡪 PS + choline
2. PE + Ser 🡪 PS + EA

Substitute for Ser and PC/PE; remove choline/EA product.

- - **17.43AcCoA + 22.43ATP + 32.38NADPH + 1.52O_2_ + DHAP + 3PG + NH_4_^+^_α-C_** 🡪 **PS**

1. **Phosphotidylinositol Synthesis**
   1. CDP-DAG + inositol 🡪 PI + CMP + Pi
      1. Inositol can come from G6P
      2. Remove CMP and P_i_ products
      3. Substitute for CDP-DAG
   2. **17.43AcCoA + 21.43ATP + 32.38NADPH + 1.52O_2_ + DHAP + (-1)NAD^+^ + G6P** 🡪 **PI**
2. **Cardiolipin synthesis**

- 2CDP-DAG + DHAP + NADH 🡪 CL + 2CMP + P_i_
  - Rearrange NAD^+^ term
  - Remove CMP ad P_i_ products
  - Substitute for CDP-DAG
- 2CDP-DAG + DHAP + (-1)NAD^+^ 🡪 CL

**3DHAP + 34.86AcCoA + 42.86ATP + 64.76NADPH + 3.04O_2_ + (-3)NAD^+^ 🡪 CL**

1. **Cholesterol synthesis**
   1. Assume all NAD(P)H terms are NADPH
   2. Many byproducts are not shown for simplicity
   3. Multiple possible biosynthetic routes, but each have similar costs; given substrate requirements are representative.
2. Mevalonate synthesis

- 3AcCoA + 2NADPH 🡪 Mev + 3CoA
  - Remove CoA term
- 3AcCoA + 2NADPH 🡪 Mev

1. Activated isoprene synthesis from mevalonate

- Mev + 3ATP 🡪 DMPP + 3ADP + P_i_ + CO_2_
  - Remove CO_2_ term
  - Simplify ATP notation
  - Substitute for Mev
- Mev + 3ATP 🡪 DMPP
- 3AcCoA + 2NADPH + 3ATP 🡪 DMPP

1. Squalene synthesis from activated isoprenes

- 6DMPP + NADPH 🡪 Squalene + NADP^+^ + 2PP_i_
  - Reduce terms
  - Substitute for DMPP
- 18AcCoA + 13NADPH + 18ATP 🡪 Squalene

1. Cyclization of squalene to cholesterol

- Squalene + 18NADPH + 11O_2_ 🡪 Chol + formate
  - Formate can be converted to CHO-THF, but 1C formation is excluded here for simplicity. However, it is considered in section on serine/glycine/1C metabolism.
  - Substitute for squalene.
- **18AcCoA + 31NADPH + 18ATP + 11O_2_ 🡪 Chol**

1. **Sphingomyelin Synthesis**
   1. Palm-CoA + Ser + FA-CoA + 2NADPH + O_2_ + PC 🡪 Sphing + CoA + CO_2_ + 1,2-DAG
      1. Substitute for Palm-CoA, FA-CoA, and Ser
      2. PC is considered equivalent to 1,2-DAG, 3ATP, and choline (see Phosphatidylcholine Synthesis)
      3. Remove CoA and CO_2_ terms
   2. **16.715AcCoA + 21.715ATP + 32.19NADPH + 1.76O_2_ + 3PG + NH_4_^+^_α-C_ + NAD^+^ + choline 🡪 Sphing**

**Polysaccharides**

**Glycogen synthesis**

The term reported by Sheikh et al., 2005 actually reports the number of mmol glucose monomer units in the measured glycogen. For a polysaccharide consisting of glucose monomers:

1. Glucose isomerization

- G6P 🡪 G1P

1. Glucose activation

- G1P + UTP 🡪 UDP-glc + PP_i_
  - UDP group transfer is considered equivalent to ATP hydrolysis; simplify notation
- G1P + ATP 🡪 UDP-glc

1. Glucose addition to polymer of n-1 monomer units

- UDP-glc + glycogen_n-1_ 🡪 glycogen_n_ + UDP
  - Remove UDP term
  - Substitute for UDP-glc and G1P
- UDP-glc + glycogen_n-1_ 🡪 glycogen_n_
- G6P + ATP + glycogen_n-1_ 🡪 glycogen_n_

1. Reduction to monomer precursors

Since mmol are given as glucose monomer units, and each monomer must come from G6P, for a glycogen polymer of n monomers:

- **nG6P + nATP** 🡪 **glycogen_­n_**

**Large Supplementary Tables**

**Table S9. Metabolites included in the stoichiometric matrix.**

| **Metabolite** | **Name** | **Abbreviation** |
| --- | --- | --- |
|  | ***Glycolysis*** |  |
| $m_{1}$ | Glucose | Glc |
| $m_{2}$ | Glucose 6-phosphate | G6P |
| $m_{3}$ | Fructose 6-phosphate | F6P |
| $m_{4}$ | Fructose 1,6-bisphosphate | FBP |
| $m_{5}$ | Dihydroxyacetone phosphate | DHAP |
| $m_{6}$ | Glyceraldehyde 3-phosphate | GAP |
| $m_{7}$ | 1,3-Bisphosphoglycerate | BPG |
| $m_{8}$ | 3-Phosphoglyerate | 3PG |
| $m_{9}$ | 1-Phosphoglycerate | 2PG |
| $m_{10}$ | Phosphoenolpyruvate | PEP |
| $m_{11}$ | Pyruvate, cytosolic | Pyr_c_ |
| $m_{12}$ | Lactate | Lac |
|  | ***Pentose Phosphate Pathway*** |  |
| $m_{13}$ | 6-Phosphoglucono-δ-lactone | PGL |
| $m_{14}$ | Gluconate 6-phosphate | GA6P |
| $m_{15}$ | Ribulose 5-phosphate | Ru5P |
| $m_{16}$ | Ribose 5-phosphate | R5P |
| $m_{17}$ | Sedoheptulose 7-phosphate | S7P |
| $m_{18}$ | Erythrose 4-phosphate | E4P |
| $m_{19}$ | Xylulose 5-phosphate | Xu5P |
|  | ***Mitochondrial Tricarboxylic Acid Cycle*** |  |
| $m_{20}$ | Pyruvate, mitochondrial | Pyr_m_ |
| $m_{21}$ | Acetyl-coenzyme A, mitochondrial | AcCoA_m_ |
| $m_{22}$ | Oxaloacetate, mitochondrial | Oaa_m_ |
| $m_{23}$ | Citrate, mitochondrial | Cit_m_ |
| $m_{24}$ | Isocitrate, mitochondrial | ICit_m_ |
| $m_{25}$ | α-Ketoglutarate, mitochondrial | αKG_m_ |
| $m_{26}$ | Succinyl-coenzyme A | SucCoA |
| $m_{27}$ | Succinate | Suc |
| $m_{28}$ | Fumarate, mitochondrial | Fum_m_ |
| $m_{29}$ | Malate, mitochondrial | Mal_m_ |
|  | ***Cytosolic Tricarboxylic Acid Cycle*** |  |
| $m_{30}$ | Acetyl-coenzyme A, cytosolic | AcCoA_c_ |
| $m_{31}$ | Oxaloacetate, cytosolic | Oaa_c_ |
| $m_{32}$ | Citrate, cytosolic | Cit_c_ |
| $m_{33}$ | Isocitrate, cytosolic | ICit_c_ |
| $m_{34}$ | α-Ketoglutarate, cytosolic | αKG_c_ |
| $m_{35}$ | Fumarate, cytosolic | Fum_c_ |
| $m_{36}$ | Malate, cytosolic | Mal_c_ |
|  | ***Amino Acids*** |  |
| $m_{37}$ | Alanine | Ala |
| $m_{38}$ | Arginine | Arg |
| $m_{39}$ | Asparagine | Asn |
| $m_{40}$ | Aspartate, cytosolic | Asp_c_ |
| $m_{41}$ | Aspartate, mitochondrial | Asp­_m_ |
| $m_{42}$ | Cysteine | Cys |
| $m_{43}$ | Glutamine, cytosolic | Gln_c_ |
| $m_{44}$ | Glutamine, mitochondrial | Gln_m_ |
| $m_{45}$ | Glutamate, cytosolic | Glu_c_ |
| $m_{46}$ | Glutamate, mitochondrial | Glu_m_ |
| $m_{47}$ | Glycine, cytosolic | Gly_c_ |
| $m_{48}$ | Glycine, mitochondrial | Gly_m_ |
| $m_{49}$ | Histidine | His |
| $m_{50}$ | Isoleucine | Ile |
| $m_{51}$ | Leucine | Leu |
| $m_{52}$ | Lysine | Lys |
| $m_{53}$ | Methionine | Met |
| $m_{54}$ | Phenylalanine | Phe |
| $m_{55}$ | Proline | Pro |
| $m_{56}$ | Serine, cytosolic | Ser_c_ |
| $m_{57}$ | Serine, mitochondrial | Ser_m_ |
| $m_{58}$ | Threonine | Thr |
| $m_{59}$ | Tryptophan | Trp |
| $m_{60}$ | Tyrosine | Tyr |
| $m_{61}$ | Valine | Val |
|  | ***Miscellaneous*** |  |
| $m_{62}$ | Carbon dioxide | CO_2_ |
| $m_{63}$ | Oxygen | O_2_ |
| $m_{64}$ | Reactive oxygen species (e.g. superoxide) | ROS |
| $m_{65}$ | Choline | Choline |
| $m_{66}$ | Ethanolamine | ethanolamine |
| $m_{67}$ | Inositol | Inositol |
| $m_{68}$ | Glycogen | glycogen |
|  | ***Amino Acid Synthesis Intermediates*** |  |
| $m_{69}$ | Ammonium | NH_4_^+^ |
| $m_{70}$ | S-Adenosyl methionine | SAM |
| $m_{71}$ | S-Adenosyl homocysteine | SAhCys |
| $m_{72}$ | Homocysteine | homoCys |
| $m_{73}$ | Cystathionine | Cystathio |
| $m_{74}$ | α-Ketobutyrate, cytosolic | αKb_c_ |
| $m_{75}$ | α-Ketobutyrate, mitochondrial | αKb_m_ |
| $m_{76}$ | Propionyl coenzyme A | PropCoA |
| $m_{77}$ | (S)-Methylmalonyl coenzyme A | SMeMalCoA |
| $m_{78}$ | (R)-Methylmalonyl coenzyme A | RMeMalCoA |
| $m_{79}$ | Phosphohydroxypyruvate | PHPyr |
| $m_{80}$ | Phosphoserine | PSer |
| $m_{81}$ | Tetrahydrofolate, cytosolic | THF_c_ |
| $m_{82}$ | Tetrahydrofolate, mitochondrial | THF_m_ |
| $m_{83}$ | Methylene-tetrahydrofolate, cytosolic | CH_2_THF_c_ |
| $m_{84}$ | Methylene-tetrahydrofolate, mitochondrial | CH_2_THF_m_ |
| $m_{85}$ | Formyl-tetrahydrofolate, cytosolic | CHOTHF_c_ |
| $m_{86}$ | Formyl-tetrahydrofolate, mitochondrial | CHOTHF_m_ |
| $m_{87}$ | Formate, cytosolic | formate_c_ |
| $m_{88}$ | Formate, mitochondrial | formate_m_ |
| $m_{89}$ | Folate | Folate |
| $m_{90}$ | Dihydrofolate | DHF |
| $m_{91}$ | Methyl-tetrahydrofolate | CH_3_THF |
| $m_{92}$ | Pyrolline 5-carboxylate | P5C |
|  | ***Nucleotide Synthesis Metabolites*** |  |
| $m_{93}$ | Adenosine triphosphate | ATP |
| $m_{94}$ | Cytidine triphosphate | CTP |
| $m_{95}$ | Guanosine triphosphate | GTP |
| $m_{96}$ | Uridine triphosphate | UTP |
| $m_{97}$ | Deoxyadenosine triphosphate | dATP |
| $m_{98}$ | Deoxycytidine triphosphate | dCTP |
| $m_{99}$ | Deoxyguanosine triphosphate | dGTP |
| $m_{100}$ | Deoxythymidine triphosphate | dTTP |
|  | ***Lipid Synthesis Metabolites*** |  |
| $m_{111}$ | Fatty acyl-coenzyme A | FACoA |
| $m_{112}$ | Palmitoyl-coenzyme A | PalmCoA |
| $m_{113}$ | 1,2-diacylglycerol | DAG |
| $m_{114}$ | Cytidine diphosphate diacylglycerol | CDPDAG |
| $m_{115}$ | Phosphatidylcholine | PCh |
| $m_{116}$ | Phosphatidylethanolamine | PE |
| $m_{117}$ | Phosphatidylglycerol | PG |
| $m_{118}$ | Phosphatidylinositol | PI |
| $m_{119}$ | Phosphatidylserine | PS |
| $m_{120}$ | Cardiolipin | CL |
| $m_{121}$ | Sphingomyelin | Sphing |
| $m_{122}$ | Cholesterol | Chol |
|  | ***Cofactors*** |  |
| $m_{123}$ | Nicotinamide adenine dinucleotide, oxidized, cytosolic | NAD^+^_c_ |
| $m_{124}$ | Nicotinamide adenine dinucleotide, reduced, cytosolic | NADH_c_ |
| $m_{125}$ | Nicotinamide adenine dinucleotide phosphate, oxidized, cytosolic | NADP^+^_c_ |
| $m_{126}$ | Nicotinamide adenine dinucleotide phosphate, reduced, cytosolic | NADPH_c_ |
| $m_{127}$ | Nicotinamide adenine dinucleotide, oxidized, mitochondrial | NAD^+^_m_ |
| $m_{128}$ | Nicotinamide adenine dinucleotide, reduced, mitochondrial | NADH_m_ |
| $m_{129}$ | Nicotinamide adenine dinucleotide phosphate, oxidized, mitochondrial | NADP^+^_m_ |
| $m_{130}$ | Nicotinamide adenine dinucleotide phosphate, reduced, mitochondrial | NADPH_m_ |
| $m_{131}$ | Flavin adenine dinucleotide, oxidized | FAD |
| $m_{132}$ | Flavin adenine dinucleotide, reduced | FADH_2_ |
| $m_{133}$ | High-energy phosphate bond breaking (e.g. ATP → ADP + P_i_, PP_i_ → 2P_i_) | ATP |
|  | ***Biomass*** |  |
| $m_{134}$ | Protein | Protein |
| $m_{135}$ | DNA | DNA |
| $m_{136}$ | RNA | RNA |
| $m_{137}$ | Lipid | Lipid |
| $m_{138}$ | Polysaccharide | Polysacc |
| $m_{139}$ | Biomass | Biomass |
|  | ***External Metabolites*** |  |
| $m_{140}$ | Glucose, external | Glc_ext_ |
| $m_{141}$ | Glutamine, external | Gln_ext_ |
| $m_{142}$ | Lactate, external | Lac_ext_ |
| $m_{143}$ | Glutamate, external | Glu_ext_ |
| $m_{144}$ | Alanine, external | Ala_ext_ |
| $m_{145}$ | Arginine, external | Arg_ext_ |
| $m_{146}$ | Asparagine, external | Asn_ext_ |
| $m_{147}$ | Aspartate, external | Asp_ext_ |
| $m_{148}$ | Cysteine, external | Cys_ext_ |
| $m_{149}$ | Glycine, external | Gly_ext_ |
| $m_{150}$ | Histidine, external | His_ext_ |
| $m_{151}$ | Isoleucine, external | Ile_ext_ |
| $m_{152}$ | Leucine, external | Leu_ext_ |
| $m_{153}$ | Lysine, external | Lys_ext_ |
| $m_{154}$ | Methionine, external | Met_ext_ |
| $m_{155}$ | Phenylalanine, external | Phe_ext_ |
| $m_{156}$ | Proline, external | Pro_ext_ |
| $m_{157}$ | Serine, external | Ser_ext_ |
| $m_{158}$ | Threonine, external | Thr_ext_ |
| $m_{159}$ | Tryptophan, external | Trp_ext_ |
| $m_{160}$ | Tyrosine, external | Tyr_ext_ |
| $m_{161}$ | Valine, external | Val_ext_ |
| $m_{162}$ | Oxygen, external | O_2ext_ |
| $m_{163}$ | Folate, external | folate_ext_ |
| $m_{164}$ | Choline, external | choline_ext_ |
| $m_{165}$ | Ethanolamine, external | ethanolamine_ext_ |
| $m_{166}$ | Inositol, external | inositol_ext_ |
| $m_{167}$ | Ammonium, external | NH_4_^+^_ext_ |
| $m_{168}$ | Carbon dioxide, external | CO_2ext_ |

**Table S10. Reactions included in the stoichiometric matrix.**

| **Flux** | **Reaction** | **Label** |
| --- | --- | --- |
|  | ***Glycolysis*** |  |
| $v_{1}$ | Glc + ATP → G6P | HK |
| $v_{2}$ | G6P ↔ F6P | GPI |
| $v_{3}$ | F6P + ATP → FBP | PFK |
| $v_{4}$ | FBP ↔ DHAP + GAP | ALDO |
| $v_{5}$ | DHAP ↔ GAP | TPI |
| $v_{6}$ | GAP + NAD^+^_c_ ↔ BPG + NADH_c_ | GAPDH |
| $v_{7}$ | BPG → 3PG + ATP | PGK |
| $v_{8}$ | 3PG ↔ 2PG | PGAM |
| $v_{9}$ | 2PG ↔ PEP | ENO |
| $v_{10}$ | PEP → Pyr_c_ + ATP | PK |
| $v_{11}$ | Pyr_c_ ↔ Lac | LDH |
|  | ***Pentose Phosphate Pathway*** |  |
| $v_{12}$ | G6P + NADP^+^_c_ → PGL + NADPH_c_ | G6PD |
| $v_{13}$ | PGL → GA6P | PGLS |
| $v_{14}$ | GA6P + NADP^+^_c_ → Ru5P + NADPH_c_ + CO_2_ | PGD |
| $v_{15}$ | Ru5P ↔ R5P | RPIA |
| $v_{16}$ | Ru5P ↔ Xu5P | RPE |
| $v_{17}$ | R5P + Xu5P ↔ GAP + S7P | TKT1 |
| $v_{18}$ | GAP + S7P ↔ E4P + F6P | TALDO |
| $v_{19}$ | E4P + Xu5P ↔ GAP + F6P | TKT2 |
|  | ***Mitochondrial Tricarboxylic Acid Cycle + Redox Reactions*** |  |
| $v_{20}$ | Pyr_m_ + NAD^+^_m_ → AcCoA_m_ + CO_2_ + NADH_m_ | PDH |
| $v_{21}$ | AcCoA_m_ + Oaa_m_ → Cit_m_ | CS |
| $v_{22}$ | Cit_m_ ↔ ICit_m_ | ACO_m_ |
| $v_{23}$ | ICit_m_ + NAD^+^_m_ → αKG_m_ + CO_2_ + NADH_m_ | IDH_NADm_ |
| $v_{24}$ | ICit_m_ + NADP^+^_m_ ↔ αKG_m_ + CO_2_ + NADPH_m_ | IDH_NADPm_ |
| $v_{25}$ | αKG_m_ + NAD_m_ → SucCoA + CO_2_ + NADH_m_ | OGDH |
| $v_{26}$ | SucCoA ↔ Suc + ATP | SUCL |
| $v_{27}$ | Suc + FAD → Fum_m_ + FADH_2_ | SDH |
| $v_{28}$ | Fum_m_ ↔ Mal_m_ | FH_m_ |
| $v_{29}$ | Mal_m_ + NAD^+^_m_ ↔ Oaa_m_ + NADH_m_ | MDH_m_ |
| $v_{30}$ | Pyr_m_ + CO_2_ + ATP → Oaa_m_ | PC |
| $v_{31}$ | Glu_m_ + NAD^+^_m_ ↔ αKG_m_ + NADH_m_ + NH_4_^+^ | GDH_NAD_ |
| $v_{32}$ | Glu_m_ + NADP^+^_m_ ↔ αKG_m_ + NADPH_m_ + NH_4_^+^ | GDH_NADP_ |
| $v_{33}$ | Gln_m_ → Glu_m_ + NH_4_^+^ | GLS |
| $v_{34}$ | Mal_m_ + NAD^+^_m_ → Pyr_m_ + CO_2_ + NADH_m_ | ME_NADm_ |
| $v_{35}$ | Mal_m_ + NADP^+^_m_ → Pyr_m_ + CO_2_ + NADPH_m_ | ME_NADPm_ |
| $v_{36}$ | NAD^+^_m_ + NADPH_m_ ↔ NADH_m_ + NADP^+^_m_ | NNT |
|  | ***Cytosolic Tricarboxylic Acid Cycle Reactions*** |  |
| $v_{37}$ | Cit_c_ + ATP → Oaa_c_ + AcCoA_c_ | ACL |
| $v_{38}$ | Cit_c_ ↔ ICit_c_ | ACO_c_ |
| $v_{39}$ | ICit_c_ + NADP^+^_c_ ↔ αKG_c_ + CO_2_ + NADPH_c_ | IDH_c_ |
| $v_{40}$ | Fum_c_ ↔ Mal_c_ | FH_c_ |
| $v_{41}$ | Mal_c_ + NAD^+^_c_ ↔ Oaa_c_ + NADH_c_ | MDH_c_ |
| $v_{42}$ | Mal_c_ + NADP^+^_c_ → Pyr_c_ + CO_2_ + NADPH_c_ | ME_c_ |
|  | ***Intercompartmental Transport*** |  |
| $v_{43}$ | Gln_m_ ↔ Gln_c_ | Gln_t_ |
| $v_{44}$ | Glu_m_ ↔ Glu_c_ | Glu_t_ |
| $v_{45}$ | Mal_m_ ↔ Mal_c_ | Mal_t_ |
| $v_{46}$ | Cit_m_ + Mal_c_ ↔ Cit_c_ + Mal_m_ | Cit-Mal_t_ |
| $v_{47}$ | Pyr_m_ ↔ Pyr_c_ | Pyr_t_ |
| $v_{48}$ | Mal_c_ + αKG_m_ ↔ Mal_m_ + αKG_c_ | αKG-Mal_t_ |
| $v_{49}$ | Glu_c_ + Asp_m_ ↔ Glu_m_ + Asp_c_ | Glu-Asp_t_ |
|  | ***External Fluxes*** |  |
| $v_{50}$ | Glc_ext_ → Glc | Glc_x_ |
| $v_{51}$ | Gln_ext_ → Gln_c_ | Gln_x_ |
| $v_{52}$ | Lac → Lac_ext_ | Lac_x_^†^ |
| $v_{53}$ | Glu_c_ → Glu_ext_ | Glu_prod_ |
| $v_{54}$ | Ala → Ala_ext_ | Ala_prod_ |
| $v_{55}$ | Arg_ext_ ↔ Arg | Arg_x_ |
| $v_{56}$ | Asn → Asn_ext_ | Asn_prod_ |
| $v_{57}$ | Asp_c_ → Asp_ext_ | Asp_prod_ |
| $v_{58}$ | Cys_ext_ → Cys | Cys_cons_ |
| $v_{59}$ | Cys → Cys_ext_ | Cys_prod_ |
| $v_{60}$ | Gly_ext_ → Gly_c_ | Gly_cons_ |
| $v_{61}$ | Gly_c_ → Gly_ext_ | Gly_prod_ |
| $v_{62}$ | His_ext_ ↔ His | His_x_ |
| $v_{63}$ | Ile_ext_ ↔ Ile | Ile_x_ |
| $v_{64}$ | Leu_ext_ ↔ Leu | Leu_x_ |
| $v_{65}$ | Lys_ext_ ↔ Lys | Lys_x_ |
| $v_{66}$ | Met_ext_ ↔ Met | Met_x_ |
| $v_{67}$ | Phe_ext_ ↔ Phe | Phe_x_ |
| $v_{68}$ | Pro → Pro_ext_ | Pro_prod_ |
| $v_{69}$ | Ser_ext_ → Ser_c_ | Ser_cons_ |
| $v_{70}$ | Ser_c_ → Ser_ext_ | Ser_prod_ |
| $v_{71}$ | Thr_ext_ ↔ Thr | Thr_x_ |
| $v_{72}$ | Trp_ext_ ↔ Trp | Trp_x_ |
| $v_{73}$ | Tyr_ext_ ↔ Tyr | Tyr_x_ |
| $v_{74}$ | Val_ext_ ↔ Val | Val_x_ |
| $v_{75}$ | O_2ext_ ↔ 0.99O_2_ + 0.01ROS | O_2x_ |
| $v_{76}$ | folate_ext_ ↔ folate | fol_x_ |
| $v_{77}$ | choline_ext_ ↔ choline | chol_x_ |
| $v_{78}$ | ethanolamine_ext_ ↔ ethanolamine | eth_x_ |
| $v_{79}$ | inositol_ext_ ↔ inositol | inos_x_ |
| $v_{80}$ | NH_4_^+^ → NH_4_^+^_ext_ | NH_4_^+^_x_ |
| $v_{81}$ | CO_2ext_ → CO_2_ | CO_2cons_ |
| $v_{82}$ | CO_2_ → CO_2ext_ | CO_2prod_ |
|  | ***Amino Acid Synthesis + One-Carbon Metabolism*** |  |
|  | *Alanine* |  |
| $v_{83}$ | Pyr_c_ + Glu_c_ ↔ Ala + αKG_c_ | ALT |
|  | *Aspartate* |  |
| $v_{84}$ | Oaa_c_ + Glu_c_ ↔ Asp_c_ + αKG_c_ | AST_c_ |
| $v_{85}$ | Oaa_m_ + Glu_m_ ↔ Asp_m_ + αKG_m_ | AST_m_ |
|  | *Asparagine* |  |
| $v_{86}$ | Asp_c_ + Gln_c_ + 2ATP → Asn + Glu_c_ | ASNS |
|  | *Cysteine* |  |
| $v_{87}$ | Met + 3ATP → SAM | MAT |
| $v_{88}$ | SAM → SAhCys | MT |
| $v_{89}$ | SAhCys → homoCys | AHCY |
| $v_{90}$ | Ser_c_ + homoCys → Cystathio | CBS |
| $v_{91}$ | Cystathio → Cys + NH_4_^+^ + αKb_c_ | CTH |
| $v_{92}$ | αKb_c_ → αKb_m_ | αKb_t_ |
| $v_{93}$ | αKb_m_ + NAD^+^_m_ → PropCoA + NADH_m_ + CO_2_ | BCKDH |
| $v_{94}$ | PropCoA + ATP + CO_2_ → SMeMalCoA | PCC |
| $v_{95}$ | SMeMalCoA → RMeMalCoA | MCEE |
| $v_{96}$ | RMeMalCoA → SucCoA | MUT |
|  | *Glycine/Serine Production* |  |
| $v_{97}$ | 3PG + NAD^+^_c_ → PHPyr + NADH_c_ | PHGDH |
| $v_{98}$ | PHPyr + Glu_c_ → PSer + αKG_c_ | PSAT |
| $v_{99}$ | PSer → Ser_c_ | PSPH |
| $v_{100}$ | Ser_c_ + THF_c_ ↔ Gly_c_ + CH_2_THF_c_ | SHMT_c_ |
| $v_{101}$ | Ser_m_ + THF_m_ ↔ Gly_m_ + CH_2_THF_m_ | SHMT_m_ |
|  | *One Carbon/Folate Metabolism* |  |
| $v_{102}$ | folate + NADPH_c_ ↔ DHF + NADP^+^_c_ | DHFR_1_ |
| $v_{103}$ | DHF + NADPH_c_ ↔ THF_c_ + NADP^+^_c_ | DHFR_2_ |
| $v_{104}$ | formate_c_ + THF_c_ + ATP → CHOTHF_c_ | MTHFD_c1_ |
| $v_{105}$ | formate_m_ + THF_m_ + ATP → CHOTHF_m_ | MTHFD_m1_ |
| $v_{106}$ | CHOTHF_c_ + NADPH_c_ ↔ CH_2_THF_c_ + NADP^+^_c_ | MTHFD_c2_ |
| $v_{107}$ | CHOTHF_m_ + NADPH_m_ ↔ CH_2_THF_m_ + NADP^+^_m_ | MTHFD_m2_ |
| $v_{108}$ | CHOTHF_c_ → formate_c_ + THF_c_ | ALDH1L_c1_ |
| $v_{109}$ | CHOTHF_m_ → formate_m_ + THF_m_ | ALDH1L_m1_ |
| $v_{110}$ | CHOTHF_c_ + NADP^+^_c_ → CO_2_ + NADPH_c_ | ALDH1L_c2_ |
| $v_{111}$ | CHOTHF_m_ + NADP^+^_m_ → CO_2_ + NADPH_m_ | ALDH1L_m2_ |
| $v_{112}$ | CH_2_THF_c_ + NADPH_c_ → CH_3_THF + NADP^+^_c_ | MTHFR |
| $v_{113}$ | homoCys + CH_3_THF → Met + THF_c_ | MetSalv |
| $v_{114}$ | Gly_m_ + THF_m_ → CH_2_THF_m_ + CO_2_ + NH_4_^+^ | GlyClv |
|  | *Glycine/Serine/One Carbon Transport* |  |
| $v_{115}$ | Ser_m_ ↔ Ser_c_ | Ser_t_ |
| $v_{116}$ | Gly_m_ ↔ Gly_c_ | Gly_t_ |
| $v_{117}$ | THF_c_ ↔ THF_m_ | THF_t_ |
| $v_{118}$ | formate_c_ ↔ formate_m_ | form_t_ |
|  | *Proline* |  |
| $v_{119}$ | Glu_c_ + ATP + NADPH_c_ → P5C + NADP^+^_c_ | P5CS |
| $v_{120}$ | P5C + NADPH_c_ → Pro + NADP^+^_c_ | P5CR_NADP_ |
| $v_{121}$ | P5C + NADH_c_ → Pro + NAD^+^_c_ | P5CR_NAD_ |
|  | *Nucleotide Synthesis* |  |
| $v_{122}$ | R5P + 2Gln_c_ + Gly_c_ + 2CHOTHF_c_ + 2Asp_c_ + 9ATP + CO_2_ → ATP_RNA_ + 2Glu_c_ + 2Fum_c_ + 2THF_c_ | ATP_RNA_ |
| $v_{123}$ | R5P + 3Gln_c_ + Gly_c_ + 2CHOTHF_c_ + Asp_c_ + 10ATP + CO_2_ + NAD^+^_c_ → GTP + 3Glu_c_ + Fum_c_ + 2THF_c_ + NADH_c_ | GTP |
| $v_{124}$ | Gln_c_ + Asp_c_ + R5P + 5.5ATP → UTP + Glu_c_ | UTP |
| $v_{125}$ | 2Gln_c_ + Asp_c_ + R5P + 6.5ATP → CTP + 2Glu_c_ | CTP |
| $v_{126}$ | R5P + 2Gln_c_ + Gly_c_ + 2CHOTHF_c_ + 2Asp_c_ + 9ATP + CO_2_ + NADPH_c_ → dATP + 2Glu_c_ + 2Fum_c_ + 2THF_c_ + NADP^+^_c_ | dATP |
| $v_{127}$ | R5P + 3Gln_c_ + Gly_c_ + 2CHOTHF_c_ + Asp_c_ + 10ATP + CO_2_ + NAD^+^_c_ + NADPH_c_ → dGTP + 3Glu_c_ + Fum_c_ + 2THF_c_ + NADH_c_ + NADP^+^_c_ | dGTP |
| $v_{128}$ | 2Gln_c_ + Asp_c_ + R5P + 6.5ATP + NADPH_c_ → dCTP + 2Glu_c_ + NADP^+^_c_ | dCTP |
| $v_{129}$ | Gln_c_ + Asp_c_ + R5P + 7.5ATP + NADPH_c_ + CH_2_THF_c_ → dTTP + Glu_c_ + NADP^+^_c_ + DHF | dTTP |
|  | *Lipid Synthesis* |  |
| $v_{130}$ | 8.715AcCoA_c_ + 9.715ATP + 16.19NADPH_c_ + 0.76O_2_ → FACoA + 16.19NADP^+^_c_ | FACoA |
| $v_{131}$ | 8AcCoA_c_ + 9ATP + 14NADPH_c_ → PalmCoA + 14NADP^+^_c_ | PalmCoA |
| $v_{132}$ | DHAP + NADH_c_ + 2FACoA → DAG + NAD^+^_c_ | DAG |
| $v_{133}$ | DHAP + NADH_c_ + 2FACoA + 2ATP → CDPDAG + NAD^+^_c_ | CDP-DAG |
| $v_{134}$ | choline + 3ATP + DAG → PCh | PChol |
| $v_{135}$ | ethanolamine + 3ATP + DAG → PE | PEth |
| $v_{136}$ | DHAP + NADH_c_ + 2ATP + DAG → PG + NAD^+^_c_ | PGly |
| $v_{137}$ | Ser_c_ + 3ATP + DAG → PS | PSer |
| $v_{138}$ | CDPDAG + inositol → PI | PInos |
| $v_{139}$ | 2CDPDAG + DHAP + NADH_c_ → CL + NAD^+^_c_ | CL |
| $v_{140}$ | 18AcCoA + 31NADPH_c_ + 11O_2_ + 18ATP → Chol + 31NADP^+^_c_ + 8CO_2_ + formate_c_ | Chol |
| $v_{141}$ | PalmCoA + FACoA + Ser_c_ + 2NADPH_c_ + PCh + O_2_ → Sphing + 2NADP^+^_c_ + DAG | Sphing |
|  | *Glycogen Synthesis* |  |
| $v_{142}$ | G6P + ATP → glycogen | Glyco |
|  | *Biomass* |  |
| $v_{143}$ | 1800Ala + 1131Arg + 1077Asp_c_ + 864Asn + 435Cys + 966Gln_c_ + 1158Glu_c_ + 1614Gly_c_ + 429His + 972Ile + 1692Leu + 1710Lys + 414Met + 657Phe + 939Pro + 1290Ser_c_ + 1158Thr + 132Trp + 546Tyr + 1248Val + 87118.992ATP → Protein | Protein |
| $v_{144}$ | 44.4dATP + 29.7dCTP + 29.7dGTP + 44.4dTTP + 203.3304ATP → DNA | DNA |
| $v_{145}$ | 99ATP_RNA_ + 165.3CTP + 187.2GTP + 99UTP + 220.2ATP → RNA | RNA |
| $v_{146}$ | 54Chol + 207PCh + 78PE + 30PI + 9PS + 3PG + 9CL + 24Sphing → Lipid | Lipid |
| $v_{147}$ | 837glycogen → Polysacc | Polysacc |
| $v_{148}$ | Protein + RNA + DNA + Lipid + Polysacc → Biomass | Biomass^†^ |
|  | *Electron Transport Chain, Redox Control, and Maintenance* |  |
| $v_{149}$ | O_2_ + NADH_m_ → NAD^+^_m_ + 2.5ATP | ETC_NADH_ |
| $v_{150}$ | O_2_ + FADH_2_ → FAD + 1.5ATP | ETC_FADH2_ |
| $v_{151}$ | ROS + NADPH_m_ → NADP^+^_m_ | AntiOx |
| $v_{152}$ | ATP -> Ø | Maint^†^ |

† indicates constrained flux (“Lac_x_” lower bound set to 418 fmol cell^-1^ h^-1^, “Biomass” equal to 0.0289 h^-1^, and “Maint” equal to 558 fmol cell^-1^ h^-1^)

**Table S11.** **Complete flux distributions from metformin treatment simulations.**

|  | **Inhibition of NADH Oxidation by ETC** | | | | | |
| --- | --- | --- | --- | --- | --- | --- |
| **Flux** | **0%** | **20%** | **40%** | **60%** | **80%** | **100%** |
| HK | 244.87 | 289.49 | 336.52 | 383.55 | 430.58 | 477.61 |
| GPI | 241.97 | 286.58 | 333.61 | 380.64 | 427.67 | 474.7 |
| PFK | 240.36 | 284.97 | 332 | 379.03 | 426.06 | 473.09 |
| ALDO | 240.36 | 284.97 | 332 | 379.03 | 426.06 | 473.09 |
| TPI | 239.12 | 283.73 | 330.76 | 377.79 | 424.82 | 471.85 |
| GAPDH | 478.67 | 567.89 | 661.95 | 756.01 | 850.07 | 944.13 |
| PGK | 478.67 | 567.89 | 661.95 | 756.01 | 850.07 | 944.13 |
| PGAM | 472.22 | 567.89 | 661.95 | 756.01 | 850.07 | 944.13 |
| ENO | 472.22 | 567.89 | 661.95 | 756.01 | 850.07 | 944.13 |
| PK | 472.22 | 567.89 | 661.95 | 756.01 | 850.07 | 944.13 |
| LDH | 418 | 514.21 | 614.6 | 714.99 | 815.39 | 915.78 |
| G6PD | 1.34E-09 | 8.45E-24 | -9.7E-26 | 2.8E-17 | 3.09E-21 | 4.47E-18 |
| PGLS | 1.34E-09 | 1.13E-22 | 3.48E-20 | 2.61E-17 | 3.85E-21 | -6.4E-19 |
| PGD | 1.34E-09 | 5.58E-23 | 4.06E-20 | 2.58E-17 | 4.33E-21 | 6.1E-18 |
| RPIA | 1.6154 | 1.6154 | 1.6154 | 1.6154 | 1.6154 | 1.6154 |
| RPE | -1.6154 | -1.6154 | -1.6154 | -1.6154 | -1.6154 | -1.6154 |
| TKT1 | -0.8077 | -0.8077 | -0.8077 | -0.8077 | -0.8077 | -0.8077 |
| TALDO | -0.8077 | -0.8077 | -0.8077 | -0.8077 | -0.8077 | -0.8077 |
| TKT2 | -0.8077 | -0.8077 | -0.8077 | -0.8077 | -0.8077 | -0.8077 |
| PDH | 51.652 | 47.897 | 41.563 | 35.229 | 28.895 | 22.561 |
| CS | 51.652 | 47.897 | 41.563 | 35.229 | 28.895 | 22.561 |
| ACO_m_ | -18.936 | -25.916 | -32.25 | -38.584 | -44.918 | -51.263 |
| IDH_NADm_ | 3.04E-11 | -1.6E-21 | -1.2E-18 | 3.32E-17 | -5.4E-20 | 8.5E-06 |
| IDH_NADPm_ | -18.936 | -25.916 | -32.25 | -38.584 | -44.918 | -51.263 |
| OGDH | 37.621 | 30.64 | 24.306 | 17.972 | 11.638 | 5.3042 |
| SUCL | 37.621 | 30.64 | 24.306 | 17.972 | 11.638 | 5.3042 |
| SDH | 37.621 | 30.64 | 24.306 | 17.972 | 11.638 | 5.3042 |
| FH_m_ | 37.621 | 30.64 | 24.306 | 17.972 | 11.638 | 5.3042 |
| MDH_m_ | 99.061 | 81.875 | 69.207 | 56.539 | 43.871 | 31.214 |
| PC | -1.1E-18 | 4.96E-22 | -1.1E-24 | 2.38E-17 | 5.74E-20 | -2.2E-11 |
| GDH_NAD_ | -14.22 | -17.018 | -16.258 | -18.199 | -20.549 | -22.528 |
| GDH_NADP_ | 3.4608 | 9.4836 | 8.7231 | 10.664 | 13.014 | 14.993 |
| GLS | 10.76 | 7.5346 | 7.5346 | 7.5346 | 7.5346 | 7.5346 |
| ME_NADm_ | 7.85E-19 | 4.8E-10 | 7.3E-27 | -3.8E-17 | 1.43E-20 | 7.38E-19 |
| ME_NADPm_ | -3.3E-18 | 6.12E-05 | -2.7E-27 | 1.33E-17 | -1E-20 | -2.5E-11 |
| NNT | -17.663 | -18.234 | -24.949 | -28.961 | -32.566 | -36.551 |
| ACL | 25.616 | 25.616 | 25.616 | 25.616 | 25.616 | 25.616 |
| ACO_c_ | 44.972 | 48.197 | 48.197 | 48.197 | 48.197 | 48.207 |
| IDH_c_ | 44.972 | 48.197 | 48.197 | 48.197 | 48.197 | 48.207 |
| FH_c_ | 1.7468 | 1.7468 | 1.7468 | 1.7468 | 1.7468 | 1.7468 |
| MDH_c_ | -63.373 | -49.943 | -43.608 | -37.274 | -30.94 | -24.617 |
| ME_c_ | 3.6796 | 0.45459 | 0.45465 | 0.45466 | 0.45466 | 0.45465 |
| Gln_t_ | -10.76 | -7.5346 | -7.5346 | -7.5346 | -7.5346 | -7.5346 |
| Glu_t_ | 21.519 | 15.069 | 15.069 | 15.069 | 15.069 | 15.069 |
| Mal_t_ | -10.76 | -7.5346 | -7.5346 | -7.5346 | -7.5346 | -7.5346 |
| Cit-Mal_t_ | 70.588 | 73.813 | 73.813 | 73.813 | 73.813 | 73.824 |
| Pyr_t_ | -51.652 | -47.897 | -41.563 | -35.229 | -28.895 | -22.561 |
| αKG-Mal_t_ | -19.908 | -30.113 | -36.447 | -42.781 | -49.115 | -55.449 |
| Glu-Asp_t_ | 47.408 | 33.978 | 27.644 | 21.31 | 14.976 | 8.6525 |
| Glc_x_ | 244.87 | 289.49 | 336.52 | 383.55 | 430.58 | 477.61 |
| Gln_x_ | 22.207 | 18.982 | 18.982 | 18.982 | 18.982 | 18.982 |
| Lac_x_ | 418 | 514.21 | 614.6 | 714.99 | 815.39 | 915.78 |
| Glu_prod_ | -1E-18 | 3.29E-22 | 4.73E-19 | -7.3E-17 | -2.2E-20 | -3.2E-07 |
| Ala_prod_ | -1.4E-19 | 1.21E-07 | 1.3E-07 | 2.03E-06 | 9.85E-06 | 9.18E-07 |
| Arg_x_ | 3.9223 | 3.9223 | 3.9223 | 3.9223 | 3.9223 | 3.9223 |
| Asn_prod_ | -8.4E-19 | 3.85E-22 | 2.13E-19 | 3.48E-17 | -3.2E-21 | -1.8E-07 |
| Asp_prod_ | -4.1E-19 | -2.4E-21 | 1E-11 | -2.7E-17 | -2.5E-20 | 3.05E-06 |
| Cys_cons_ | 1.5086 | 1.5086 | 1.5086 | 1.5086 | 1.5086 | 1.5086 |
| Cys_prod_ | -1E-18 | -2.8E-22 | 1.09E-28 | -2.7E-17 | -1.9E-20 | -3.5E-07 |
| Gly_cons_ | 4.3811 | 4.3811 | 4.3811 | 4.3811 | 4.3811 | 4.3811 |
| Gly_prod_ | 4.95E-19 | 1.77E-21 | -1.6E-18 | -1.4E-16 | -2E-20 | -4.6E-11 |
| His_x_ | 1.4878 | 1.4878 | 1.4878 | 1.4878 | 1.4878 | 1.4878 |
| Ile_x_ | 3.3709 | 3.3709 | 3.3709 | 3.3709 | 3.3709 | 3.3709 |
| Leu_x_ | 5.8679 | 5.8679 | 5.8679 | 5.8679 | 5.8679 | 5.8679 |
| Lys_x_ | 5.9303 | 5.9303 | 5.9303 | 5.9303 | 5.9303 | 5.9303 |
| Met_x_ | 1.4358 | 1.4358 | 1.4358 | 1.4358 | 1.4358 | 1.4358 |
| Phe_x_ | 2.2785 | 2.2785 | 2.2785 | 2.2785 | 2.2785 | 2.2785 |
| Pro_prod_ | 1.39E-18 | -1.5E-21 | 1.14E-18 | -4E-17 | 6.34E-20 | -9.9E-08 |
| Ser_cons_ | 0.604 | 7.0539 | 7.0539 | 7.0539 | 7.0539 | 7.0539 |
| Ser_prod_ | -1E-18 | -2.2E-22 | 4.73E-20 | 5.64E-17 | 9.51E-21 | 2.42E-19 |
| Thr_x_ | 4.0159 | 4.0159 | 4.0159 | 4.0159 | 4.0159 | 4.0159 |
| Trp_x_ | 0.45778 | 0.45778 | 0.45778 | 0.45778 | 0.45778 | 0.45778 |
| Tyr_x_ | 1.8935 | 1.8935 | 1.8935 | 1.8935 | 1.8935 | 1.8935 |
| Val_x_ | 4.3281 | 4.3281 | 4.3281 | 4.3281 | 4.3281 | 4.3281 |
| O_2x_ | 200.1 | 161.44 | 123.44 | 85.432 | 47.428 | 9.4236 |
| fol_x_ | -5.9E-17 | 3.3E-17 | 3.91E-16 | -3.2E-16 | 4.44E-16 | -3.2E-07 |
| chol_x_ | 0.80111 | 0.80111 | 0.80111 | 0.80111 | 0.80111 | 0.80111 |
| eth_x_ | 0.2705 | 0.2705 | 0.2705 | 0.2705 | 0.2705 | 0.2705 |
| inos_x_ | 0.10404 | 0.10404 | 0.10404 | 0.10404 | 0.10404 | 0.10404 |
| NH_4_^+^_x_ | 6.12E-06 | 1.46E-08 | -1.6E-27 | -5E-17 | 1.21E-20 | -1E-07 |
| CO_2cons_ | 4.64E-19 | 1.33E-21 | 1.33E-18 | 1.82E-17 | -4.3E-26 | -2.4E-07 |
| CO_2prod_ | 119.24 | 101.52 | 82.519 | 63.517 | 44.515 | 25.513 |
| ALT | 6.2424 | 6.2424 | 6.2424 | 6.2424 | 6.2424 | 6.2424 |
| AST_c_ | -37.756 | -24.326 | -17.992 | -11.658 | -5.3241 | 0.99926 |
| AST_m_ | 47.408 | 33.978 | 27.644 | 21.31 | 14.976 | 8.6525 |
| ASNS | 2.9964 | 2.9964 | 2.9964 | 2.9964 | 2.9964 | 2.9964 |
| MAT | 2.96E-19 | 4.8E-22 | 4.73E-19 | -1.3E-17 | 3.17E-21 | -3.9E-07 |
| MT | -1.3E-19 | 4.15E-22 | 2.84E-19 | 1.59E-27 | 7.14E-21 | -3.9E-07 |
| AHCY | -1.7E-18 | 1.49E-22 | 3.79E-19 | 8.62E-17 | 3.17E-20 | -3.9E-07 |
| CBS | 1.56E-19 | -6.2E-22 | -5.2E-19 | 3.65E-17 | -9.9E-21 | -3.9E-07 |
| CTH | 1.06E-18 | -7.7E-22 | -3.8E-19 | 1.33E-17 | -6.3E-21 | -3.9E-07 |
| aKb_t_ | 8.6E-19 | -7.8E-22 | -4.7E-19 | 8.29E-17 | -2.7E-20 | -3.9E-07 |
| BCKDH | 1.21E-18 | -8.8E-22 | -4.7E-19 | 7.79E-17 | -2.9E-20 | -3.9E-07 |
| PCC | 1.13E-18 | -9.5E-22 | -6.6E-19 | 7.79E-17 | -3.5E-20 | -3.9E-07 |
| MCEE | 1.64E-18 | -3.1E-22 | -7.1E-19 | 7.3E-17 | -3.2E-20 | -3.9E-07 |
| MUT | 4.51E-18 | -4.2E-22 | -8.3E-19 | 1.33E-16 | 1.74E-20 | -3.9E-07 |
| PHGDH | 6.4499 | 1.27E-22 | -6.5E-19 | -4.3E-17 | -5.3E-20 | -2.5E-07 |
| PSAT | 6.4499 | 8.18E-22 | -3.4E-19 | -5.5E-17 | -6.1E-20 | -2.5E-07 |
| PSPH | 6.4499 | 4.01E-23 | -9E-19 | -3E-17 | -3.3E-20 | -2.5E-07 |
| SHMT_c_ | 2.653 | 2.653 | 2.653 | 2.653 | 2.653 | 2.653 |
| SHMT_m_ | -0.18727 | -0.18727 | -0.18727 | -0.18727 | -0.18727 | -0.18727 |
| DHFR_1_ | -5.8E-17 | 3.3E-17 | 3.91E-16 | -3.4E-16 | 4.44E-16 | -3.2E-07 |
| DHFR_2_ | 0.15398 | 0.15398 | 0.15398 | 0.15398 | 0.15398 | 0.15398 |
| MTHFD_c1_ | -7.5E-19 | 1.11E-22 | -6.6E-19 | 2.11E-17 | 8.72E-21 | 9.04E-19 |
| MTHFD_m1_ | 0.18727 | 0.18727 | 0.18727 | 0.18727 | 0.18727 | 0.18727 |
| MTHFD_c2_ | -2.499 | -2.499 | -2.499 | -2.499 | -2.499 | -2.499 |
| MTHFD_m2_ | 0.18727 | 0.18727 | 0.18727 | 0.18727 | 0.18727 | 0.18727 |
| ALDH1L_c1_ | 1.25E-19 | -3E-22 | 8.52E-19 | 3.86E-17 | 1.67E-20 | -7.6E-08 |
| ALDH1L_m1_ | 1.61E-18 | -4.6E-23 | -3.3E-19 | -1.9E-17 | -1.9E-20 | 1.13E-18 |
| ALDH1L_c2_ | 1.05E-18 | 9.83E-22 | -2.8E-19 | -3.8E-17 | 3.17E-21 | -3.2E-07 |
| ALDH1L_m2_ | 1.01E-18 | 2.21E-22 | -4.7E-19 | -1.5E-17 | -3.5E-26 | 5.34E-19 |
| MTHFR | -1.5E-18 | 3.61E-21 | -1.9E-19 | 9.95E-18 | 6.98E-20 | 1.62E-19 |
| MetSalv | -7.7E-19 | 2.21E-21 | -9.1E-27 | -6.6E-18 | 4.92E-20 | 1.45E-18 |
| GlyClv | 1.67E-19 | -1.2E-22 | 9.47E-20 | 8.29E-19 | -6.3E-21 | 2.78E-06 |
| Ser_t_ | 0.18727 | 0.18727 | 0.18727 | 0.18727 | 0.18727 | 0.18727 |
| Gly_t_ | -0.18727 | -0.18727 | -0.18727 | -0.18727 | -0.18727 | -0.18728 |
| THF_t_ | -4.8E-18 | -2.4E-21 | 1.94E-18 | 2.2E-17 | 7.06E-20 | -6.6E-18 |
| form_t_ | -0.18727 | -0.18727 | -0.18727 | -0.18727 | -0.18727 | -0.18727 |
| P5CS | 3.2565 | 3.2565 | 3.2565 | 3.2565 | 3.2565 | 3.2565 |
| P5CR_NADP_ | -3.7E-19 | -1.5E-22 | 1.28E-08 | 1.31E-27 | 3.88E-25 | 0.010679 |
| P5CR_NAD_ | 3.2565 | 3.2565 | 3.2565 | 3.2565 | 3.2565 | 3.2458 |
| ATP_RNA_ | 0.34333 | 0.34333 | 0.34333 | 0.34333 | 0.34333 | 0.34333 |
| GTP | 0.64921 | 0.64921 | 0.64921 | 0.64921 | 0.64921 | 0.64921 |
| UTP | 0.34333 | 0.34333 | 0.34333 | 0.34333 | 0.34333 | 0.34333 |
| CTP | 0.57326 | 0.57326 | 0.57326 | 0.57326 | 0.57326 | 0.57326 |
| dATP | 0.15398 | 0.15398 | 0.15398 | 0.15398 | 0.15398 | 0.15398 |
| dGTP | 0.103 | 0.103 | 0.103 | 0.103 | 0.103 | 0.103 |
| dCTP | 0.103 | 0.103 | 0.103 | 0.103 | 0.103 | 0.103 |
| dTTP | 0.15398 | 0.15398 | 0.15398 | 0.15398 | 0.15398 | 0.15398 |
| FACoA | 2.4762 | 2.4762 | 2.4762 | 2.4762 | 2.4762 | 2.4762 |
| PalmCoA | 0.083232 | 0.083232 | 0.083232 | 0.083232 | 0.083232 | 0.083232 |
| DAG | 1.03 | 1.03 | 1.03 | 1.03 | 1.03 | 1.03 |
| CDP-DAG | 0.16646 | 0.16646 | 0.16646 | 0.16646 | 0.16646 | 0.16646 |
| PChol | 0.80111 | 0.80111 | 0.80111 | 0.80111 | 0.80111 | 0.80111 |
| PEth | 0.2705 | 0.2705 | 0.2705 | 0.2705 | 0.2705 | 0.2705 |
| PGly | 0.010404 | 0.010404 | 0.010404 | 0.010404 | 0.010404 | 0.010404 |
| PSer | 0.031212 | 0.031212 | 0.031212 | 0.031212 | 0.031212 | 0.031212 |
| PInos | 0.10404 | 0.10404 | 0.10404 | 0.10404 | 0.10404 | 0.10404 |
| CL | 0.031212 | 0.031212 | 0.031212 | 0.031212 | 0.031212 | 0.031212 |
| Chol | 0.18727 | 0.18727 | 0.18727 | 0.18727 | 0.18727 | 0.18727 |
| Sphing | 0.083232 | 0.083232 | 0.083232 | 0.083232 | 0.083232 | 0.083232 |
| Glyco | 2.9027 | 2.9027 | 2.9027 | 2.9027 | 2.9027 | 2.9027 |
| Protein | 0.0289 | 0.0289 | 0.0289 | 0.0289 | 0.0289 | 0.0289 |
| DNA | 0.0289 | 0.0289 | 0.0289 | 0.0289 | 0.0289 | 0.0289 |
| RNA | 0.0289 | 0.0289 | 0.0289 | 0.0289 | 0.0289 | 0.0289 |
| Lipid | 0.0289 | 0.0289 | 0.0289 | 0.0289 | 0.0289 | 0.0289 |
| Polysacc | 0.0289 | 0.0289 | 0.0289 | 0.0289 | 0.0289 | 0.0289 |
| Biomass | 0.0289 | 0.0289 | 0.0289 | 0.0289 | 0.0289 | 0.0289 |
| ETC_NADH_ | 156.45 | 125.16 | 93.87 | 62.58 | 31.29 | 1.58E-11 |
| ETC_FADH2_ | 37.621 | 30.64 | 24.306 | 17.972 | 11.638 | 5.3042 |
| AntiOx | 2.001 | 1.6144 | 1.2344 | 0.85432 | 0.47428 | 0.094236 |
| Maint | 558 | 558 | 558 | 558 | 558 | 558 |

All values in fmol cell^-1^ h^-1^ with an assumed per-cell dry weight of 360 pg.
