# Supplementary material for: Metabolic requirements for cancer cell proliferation
Source: Cancer Metab. 2016 Aug 18;4:16. doi: 10.1186/s40170-016-0156-6 (PMC4989334; doi:10.1186/s40170-016-0156-6)
Supplement: Additional file 3: Figure S1. — Mitochondrial NADH consumption and production flux predictions as functions of ETC inhibition for different nicotinamide nucleotide transhydrogenase (NNT) and glutamate dehydrogenase (GDH) conditions. Figure S2. Additional metabolic flux alterations predicted to occur in response to ETC inhibition in simulations of metformin treatment. (PDF 194 kb) [file 40170_2016_156_MOESM3_ESM.pdf]

Metabolic requirements for cancer cell proliferation

Supplementary Figures

Mark A. Keibler[1,a], Thomas M. Wasylenko[1,3,b], Joanne K. Kelleher[1,c], Othon Iliopoulos[4,5,d], Matthew G. Vander Heiden[2,e], Gregory Stephanopoulos[1,f]*

1. Department of Chemical Engineering, Massachusetts Institute of Technology, Cambridge, MA 02139, USA
2. Koch Institute for Integrative Cancer Research and Department of Biology, Massachusetts Institute of Technology, Cambridge, MA 02139, USA
3. Current address: Late Stage Process Development, Sanofi Genzyme, 31 New York Ave, Framingham, Massachusetts 01701
4. Center for Cancer Research, Massachusetts General Hospital Cancer Center, Charlestown, MA 02129, USA
5. Division of Hematology/Oncology, Department of Medicine, Massachusetts General Hospital, Boston, MA 02114, USA

Email Addresses: a. mkeibler@mit.edu, b. thomas.wasylenko@genzyme.com, c. jkk@mit.edu, d. iliopoul@helix.mgh.harvard.edu, e. mvh@mit.edu, f. gregstep@mit.edu

*Corresponding Author:

Phone: 617.253.4583

Fax: 617.253.3122

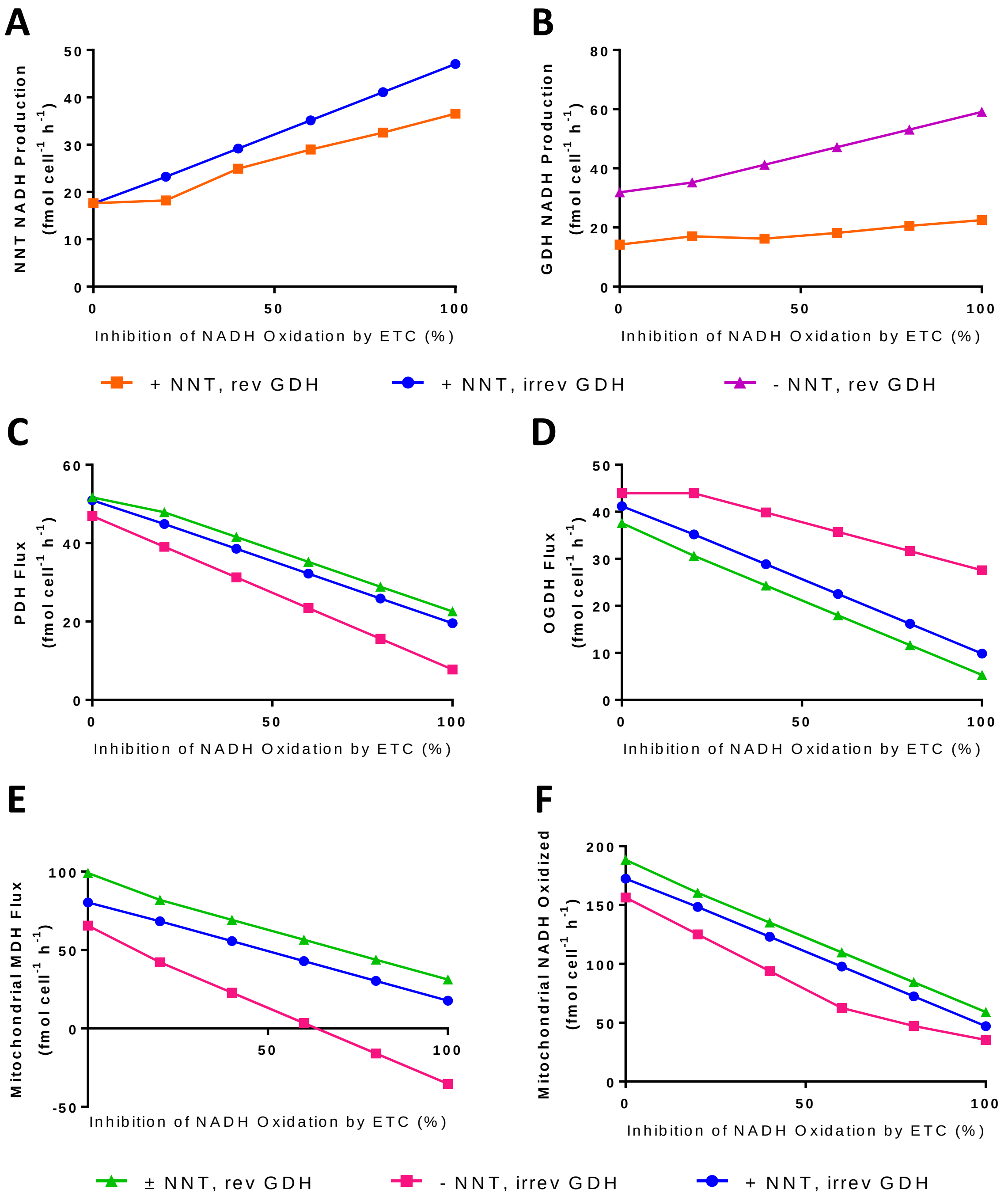

Figure S1. Mitochondrial NADH consumption and production flux predictions as functions of ETC inhibition for different nicotinamide nucleotide transhydrogenase (NNT) and glutamate dehydrogenase (GDH) conditions. (A) NADH production from NNT when GDH is either reversible (rev GDH) or irreversible (irrev GDH). NNT is included in both models. (B) NADH production from GDH when NNT is either included (+ NNT) or absent (- NNT). GDH is reversible in both models. (C) pyruvate dehydrogenase (PDH) flux, (D) oxoglutarate dehydrogenase (OGDH) flux, (E) mitochondrial malate dehydrogenase (MDH) flux, and (F) total mitochondrial NADH oxidized for different models. Both NNT-present and NNT-absent conditions gave effectively the same values when GDH was reversible (± NNT, rev GDH).

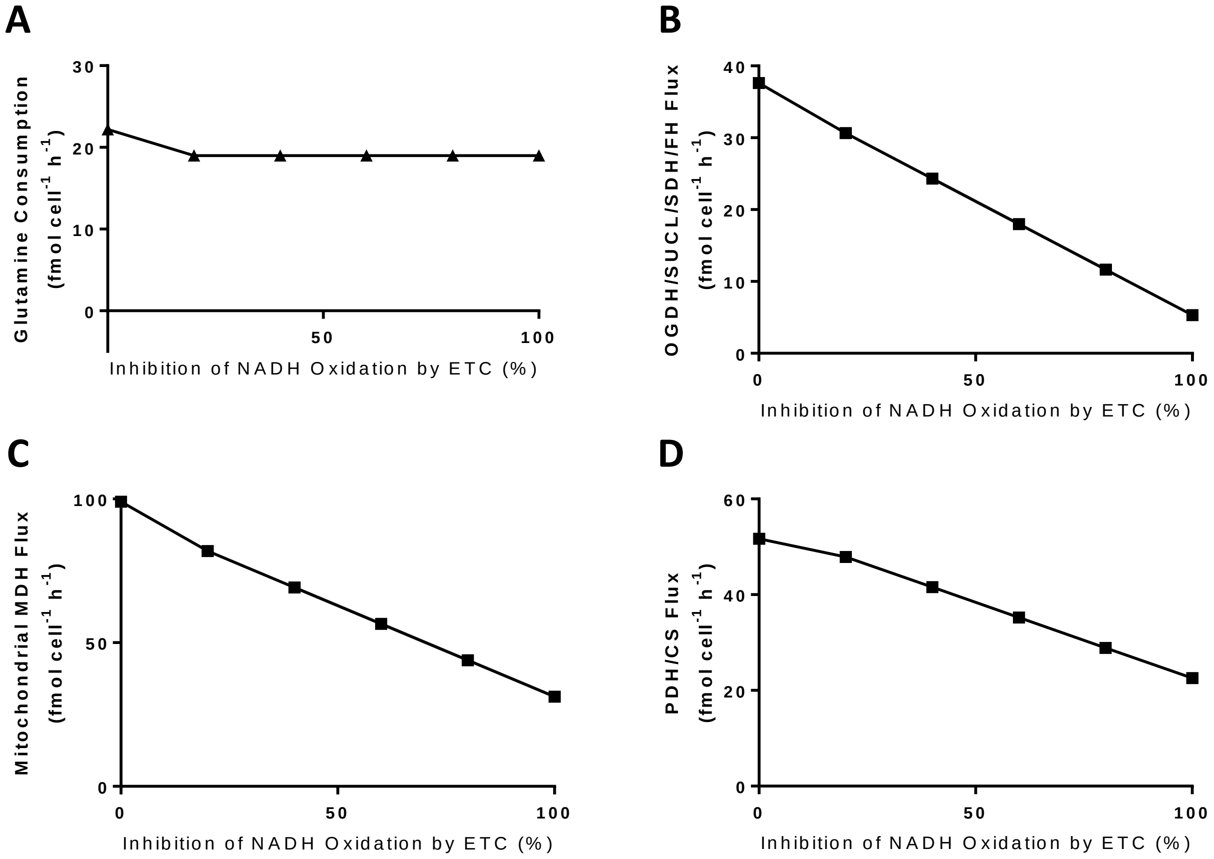

Figure S2. Additional metabolic flux alterations predicted to occur in response to ETC inhibition in simulations of metformin treatment. (A) Glutamine consumption; (B) oxoglutarate dehydrogenase (OGDH), succinate-CoA ligase (SUCL), succinate dehydrogenase (SDH), and fumarate hydratase (FH) flux; (C) mitochondrial malate dehydrogenase (MDH) flux; and (D) pyruvate dehydrogenase (PDH) and citrate synthase (CS) flux as functions of percent NADH oxidation inhibition.
